# Broad genic repression domains signify enhanced silencing of oncogenes

**DOI:** 10.1038/s41467-020-18913-8

**Published:** 2020-11-03

**Authors:** Dongyu Zhao, Lili Zhang, Min Zhang, Bo Xia, Jie Lv, Xinlei Gao, Guangyu Wang, Qingshu Meng, Yang Yi, Sen Zhu, Alin S. Tomoiaga, Min Gyu Lee, John P. Cooke, Qi Cao, Kaifu Chen

**Affiliations:** 1 Center for Bioinformatics and Computational Biology, Houston Methodist Research Institute, Houston, TX USA; 2 Center for Cardiovascular Regeneration, Department of Cardiovascular Sciences, Houston Methodist Research Institute, Houston, TX USA; 3 Department of Cardiothoracic Surgeries, Weill Cornell Medical College, Cornell University, New York, NY USA; 4 Institute for Academic Medicine, Houston Methodist Hospital, Houston, TX USA; 5 Department of Urology, Feinberg School of Medicine, Northwestern University, Chicago, IL USA; 6 Robert H. Lurie Comprehensive Cancer Center, Feinberg School of Medicine, Northwestern University, Chicago, IL USA; 7 Business Analytics, CIS & Law Department, The O’Malley School of Business Accounting, Manhattan College, Riverdale, NY USA; 8 Department of Molecular and Cellular Oncology, The University of Texas MD Anderson Cancer Center, Houston, TX USA

**Keywords:** Oncogenes, Gene silencing

## Abstract

Cancers result from a set of genetic and epigenetic alterations. Most known oncogenes were identified by gain-of-function mutations in cancer, yet little is known about their epigenetic features. Through integrative analysis of 11,596 epigenomic profiles and mutations from >8200 tumor-normal pairs, we discover broad genic repression domains (BGRD) on chromatin as an epigenetic signature for oncogenes. A BGRD is a widespread enrichment domain of the repressive histone modification H3K27me3 and is further enriched with multiple other repressive marks including H3K9me3, H3K9me2, and H3K27me2. Further, BGRD displays widespread enrichment of repressed *cis*-regulatory elements. Shortening of BGRDs is linked to derepression of transcription. BGRDs at oncogenes tend to be conserved across normal cell types. Putative tumor-promoting genes and lncRNAs defined using BGRDs are experimentally verified as required for cancer phenotypes. Therefore, BGRDs play key roles in epigenetic regulation of cancer and provide a direction for mutation-independent discovery of oncogenes.

## Abstract (web summary)

Epigenetically altered genes can have a key role in cancer pathobiology but epigenetic signatures that distinguish oncogenes are not yet known. Here, the authors identify broad genic repression domains, defined by widespread H3K27me3 modification, as an epigenetic signature to provide mutation-independent information for discovery of potential oncogenes.

## Introduction

Transcriptional control is dependent on epigenetic modifications. One modification of particular interest is the tri-methylation of lysine 27 on histone H3 (H3K27me3) catalyzed by the Polycomb Repressive Complex 2 (PRC2)^1,2^. H3K27me3 is a hallmark of heterochromatin. It is not completely clear how it functions in gene repression^3^. It maintains chromatin repression established early in *Drosophila* development^4–6^. PRC2 proteins were found mutated or deregulated in diseases including cancer^7^. The activity of PRC2 toward chromatin is largely predominant in cancer^8^. Some cancer genes were uncovered by H3K27me3 analysis^9–11^. However, the mechanism of H3K27me3 alterations induced cancer is not yet completely clear.

Recent genome sequencing efforts have successfully detected millions of genetic mutations in tens of thousands of tumors and normal samples^12,13^. However, only a small fraction of mutations have been found to involve oncogenes^14^. We are in need of alternative methods to distinguish independently which sequences are clinically important^15^. Meanwhile, there is ample evidence for clinically important genes that rarely mutate, but act as cancer drivers, e.g., due to epigenetic alterations^15^. Genes suspected of increasing the selective growth advantage of tumor cells have been categorized as either Mut-driver genes or Epi-driver genes^16^. Mut-driver genes contain a sufficient number of driver mutations to unambiguously distinguish them from other genes. Epi-driver genes are expressed aberrantly in tumors but do not frequently mutate. Unlike genetic sequences that are highly stable in a given individual, many epigenetic modifications vary with normal biological contexts. Criteria have yet to be formulated for distinguishing epigenetic changes that exert a selective growth advantage from those that do not.

In this study, we describe the discovery of broad genic repression domains (BGRD), defined by widespread H3K27me3 modification, as an epigenetic signature to provide mutation-independent information for discovery of oncogenes. We illustrate how this signature is linked to gene structure and the composition of *cis*-regulatory elements. We subsequently utilize the BGRD signature to identify oncogenes, as well as oncogenic lncRNAs followed by comprehensive verifications.

## Results

### Broad enrichment of H3K27me3 at oncogenes

**Fig. 1:**
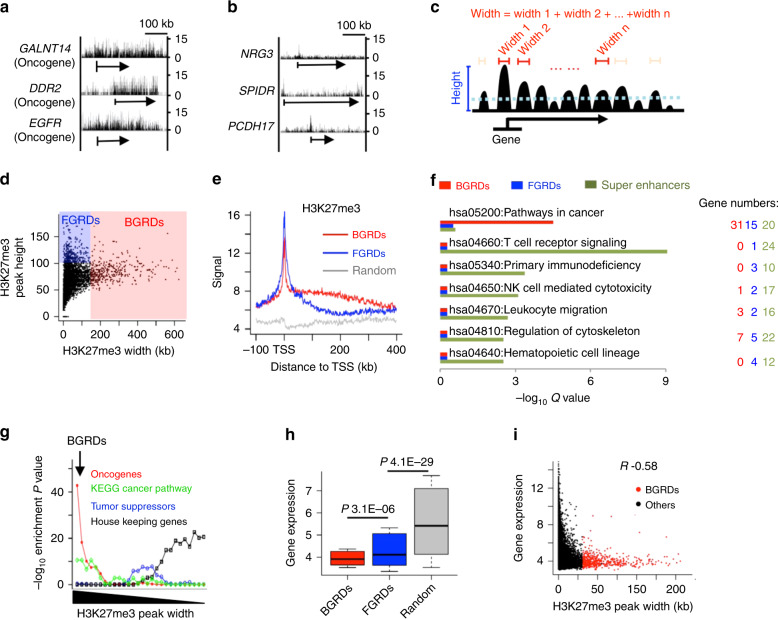
Oncogenes reside in BGRDs on chromatin in normal CD4^+^ T cell. **a**, **b** H3K27me3 ChIP-Seq signal in three BGRDs **a** and three FGRDs **b**. Arrows indicate gene loci associated with these repressive domains. Gene names were indicated at the left side. *Y*-axis scale at the right side indicates ChIP-Seq signal strength. **c** Cartoon to show the definition of width and height for H3K27me3 ChIP-Seq enrichment peaks on a gene. **d** Height plotted against width of H3K27me3 ChIP-Seq enrichment peaks at each gene. Red and blue areas indicate peaks that were defined to be BGRDs and FGRDs, respectively. **e** Average ChIP-Seq signal value for H3K27me3 plotted around TSS associated with each category of repressive domains. **f** Enrichment level of KEGG pathways in genes associated with BGRDs, FGRDs, or super enhancers. *Q* value indicates Benjamini-adjusted *P* value. **g** Enrichment level of each gene category plotted against H3K27me3 peak width. Genes were ranked by H3K27me3 width and divided into groups that each contain 1500 genes, with two neighboring groups in the rank has 500 genes in common. A dot in each curve indicates the enrichment level (*Y*-axis) of one of these groups (*X*-axis) in the oncogenes, KEGG cancer pathway genes, tumor suppressor genes, or housekeeping genes as indicated by the color legends. **h** Boxplot to show microarray expression values (*Y*-axis) of genes associated with each category of repressive domains (*Y*-axis), *n* = 500 for each category. Box plots: center line is median, boxes show first and third quartiles, whiskers extend to the most extreme data points that are no more than 1.5-fold of the interquartile range from the box. **i** Microarray expression value of each gene plotted against width of the gene’s H3K27me3 enrichment peak, with Spearman correlation coefficients indicated on top. *P* values determined by one tail Fisher’s exact test **g** or one tail Wilcoxon test **h**. Source data are provided as a Source Data file.

We initially observed that H3K27me3 spanned several hundred kilo-base pairs (kb) in human CD4^+^ T cells on oncogenes such as *GALNT14*^17^, *DDR2*^18^, and *EGFR*^19^ (Fig. 1a). In contrast, H3K27me3 covered only several kb for many other genes such as *NRG3*, *SPIDR*, and *PCDH17* (Fig. 1b). This motivated us to specifically define widths of H3K27me3 ChIP-Seq enrichment peaks on individual genes (Fig. 1c), which revealed two distinct patterns (Fig. 1d): (1) focal genic repression domains (FGRDs) whose H3K27me3 peaks are high but narrow; and (2) BGRDs whose H3K27me3 peaks display an intermediate height but are widespread. The cumulative plot of H3K27me3 width is close to an L shape (Supplementary Fig. 1a), with the turning point at 60.5 kb. We used an H3K27me3 width cutoff at 121 kb, which is twofold of the width at the turning point (Supplementary Fig. 1a), to define 500 genes associated with BGRDs. We further defined 500 genes associated with FGRDs that displayed the highest H3K27me3 peaks (Fig. 1d), and also defined 500 random control genes. H3K27me3 in BGRDs showed a skewed distribution relative to the transcription start site (TSS), i.e., a sharp enrichment peak in the promoter, followed by a long enrichment tail covering the gene body (Fig. 1e, Supplementary Fig. 1b). In contrast, H3K27me3 in FGRDs was limited to a short region around TSS. Although defined in CD4^+^ T cells, BGRD genes were not enriched in T cell pathways, but were enriched in the Pathways In Cancer (KEGG entry ID hsa05200) (Fig. 1f), a set of 330 cancer genes manually curated from literature^20^. A cancer pathway includes both oncogenes and tumor suppressors. We further analyzed oncogenes and tumor suppressors defined by TUSON using cancer mutation signatures^21^, and found BGRDs enriched in oncogenes but not tumor suppressor genes (Fig. 1g, Supplementary Fig. 1c, and Supplementary Data 1a). For comparison, super enhancer genes, well known to be cell identity genes^22,23^, were enriched in T cell pathways but not in the Pathways In Cancer (Fig. 1f). FGRDs showed no enrichment in oncogenes, tumor suppressors (Supplementary Fig. 1d and Supplementary Data 1b) or control genes (Supplementary Fig. 1e and Supplementary Data 1c). Housekeeping genes^24^ were enriched in genes that have no BGRDs (Fig. 1g, Supplementary Fig. 1c, and Supplementary Data 1a) and no FGRDs (Supplementary Fig. 1d and Supplementary Data 1b). The overlap between genes associated with BGRDs and the KEGG cancer genes or the oncogenes predicted by TUSON is larger than the overlap between the KEGG cancer genes and the oncogenes predicted by TUSON (Supplementary Fig. 1f, g). In summary, BGRDs in CD4^+^ T cell are associated with oncogenes but not with tumor suppressors or CD4^+^ T cell specific pathways.

Several other repressive histone modifications, e.g., H3K27me2 (Supplementary Fig. 1h), H3K9me2 (Supplementary Fig. 1i), and H3K9me3 (Supplementary Fig. 1j), showed similar widespread enrichment in the BGRDs defined by widespread H3K27me3. BGRDs directly defined by widespread enrichment of these modifications also showed enrichment in oncogenes (Supplementary Fig. 2a). Most of the histone modifications associated with activation of transcription displayed broad depletion in BGRDs (Supplementary Fig. 2b, c), e.g., the H3K4me1 (Supplementary Fig. 2d) and H3K4me2 (Supplementary Fig. 2e). A subset of the activating histone modifications, e.g., the H3K4me3 (Supplementary Fig. 2f), did not display broad enrichment or broad depletion in BGRDs. RNA expression is significantly lower for genes marked with BGRDs relative to genes associated with FGRDs or random domains (Fig. 1h). However, among the repressed genes, only a small subset was marked by BGRDs (Fig. 1i) or FGRDs (Supplementary Fig. 2g). Therefore, BGRDs in CD4^+^ T cell were associated with enhanced repression of oncogenes, but not with other silent genes.

We next performed a comparison between BGRD and Large Organized Chromatin K9-modification (LOCK), another type of repressive domain defined by H3K9me2 but was found not associated with H3K27me3^25^. We downloaded the published LOCKs and H3K27me3 ChIP-Seq data from the same tissue types to define BGRDs. Only 8 of the 67 LOCKs overlapped with 15 of the 3765 BGRDs in human placenta, and 14 of the 2562 LOCKs overlapped with 13 of the 2091 BGRDs in mouse brain (Supplementary Fig. 3a, b). We further compared LOCKs with the same number of the broadest BGRDs in each tissue type, and observed <6% overlap (Supplementary Fig. 3c, d). In contrast to the reported lower gene density in LOCKs when compared to random domains, BGRDs displayed 2.5-fold higher gene density (Supplementary Fig. 3e, f). There is <8% overlap between LOCK-associated and BGRD-associated genes (Supplementary Fig. 3g, h). BGRDs in these tissues were enriched with oncogenes (Supplementary Fig. 3i, j). In contrast, LOCKs showed little enrichment of oncogenes. Further analysis indicated that LOCKs were twofold broader than FGRDs but fourfold narrower than BGRDs (Supplementary Fig. 3k, l). Intriguingly, LOCK sizes are significantly larger than their associated gene sizes; in contrast, BGRDs sizes are significantly smaller than the sizes of their associated genes (Supplementary Fig. 3k, l). Another group reported Broad H3K27me3 defined by average density of H3K27me3 on gene body^1^, which is different from our calculation of H3K27me3 width on gene body. There is <5% overlap between BGRDs and the Broad H3K27me3 (Supplementary Fig. 4a). Also, BGRD is enriched with oncogenes, while the Broad H3K27me3 is not (Supplementary Fig. 4b and Supplementary Data 2). Therefore, although BGRD, LOCK, and Broad H3K27me3 are all broad, they are different in definition and regulate genes associated with different functions.

### Long genes display either BGRDs or FGRDs

**Fig. 2:**
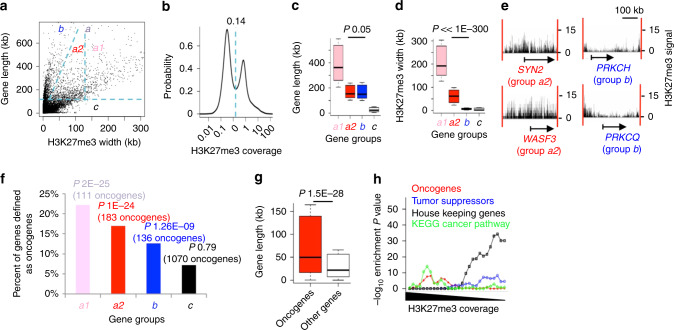
BGRD genes tend to have a long gene body, whereas only a subset of long genes has BGRDs. **a** Gene length plotted against width of H3K27me3 ChIP-Seq enrichment peaks on the gene. Dash lines indicate the boundaries for individual gene groups, *a1*(pink color), *a2*(red color), *b*(blue color), and *c*(black color). **b** The probability distribution of H3K27me3 coverage values across all genes. The dash line indicates boundary between the two peaks in the distribution. Boxplot to show gene lengths (**c**) and repression domain widths (**d**) of each gene group indicated in **a**, n(*a1*) = 500, n(*a2*) = 1080, n(*b*) = 1080, and n(*c*) = 11880. **e** H3K27me3 ChIP-Seq signal at example genes. Arrows indicate gene loci. Gene names were indicated at the bottom. *Y*-axis scale at the right side indicates ChIP-Seq signal strength. The scale of region size was indicated on top. **f** Bar plot to show percent of oncogenes in individual gene groups. The number of oncogenes and *P* value of oncogene enrichment were indicated on top of each bar. **g** Boxplot to show length of oncogenes and other genes, *n* = 500 for each group. **h** Enrichment level of each gene category plotted against H3K27me3 coverage of each gene. Coverage defined as the width of H3K27me3 ChIP-Seq enrichment peaks divided by gene length. Genes were ranked by H3K27me3 coverage and divided into groups that each contains 1500 genes, with two neighboring groups in the rank has 500 genes in common. A dot in each curve indicates the enrichment level (*Y*-axis) of one of these groups (*X*-axis) in the oncogenes, KEGG cancer pathway genes, tumor suppressor genes, or housekeeping genes as indicated by the color legends. *P* values calculated by one tail Wilcoxon test **c**, **d**, **g** or one tail Fisher’s exact test **f**, **h**. Box plots: center line is median, boxes show first and third quartiles, whiskers extend to the most extreme data points that are no more than 1.5-fold of the interquartile range from the box. Source data are provided as a Source Data file.

Because BGRDs cover both promoter and gene body (Fig. 1a, e and Supplementary Fig. 1b), we may expect that BGRD genes tend to be long genes. However, it is unclear whether the long genes always display BGRDs but not FGRDs, and whether FGRDs always appear at short genes but not at long genes. We plotted gene length against H3K27me3 breadth in the CD4^+^ T cell (Fig. 2a), and observed two distinct gene clusters that are different in their H3K27me3 coverage (H3K27me3 width divided by gene length), i.e., a high-coverage cluster and a low-coverage cluster. The boundary between the two clusters is at the coverage value 0.14 (Fig. 2b). BGRD genes are a subset (group *a1* in Fig. 2a) of the high-coverage cluster. These genes tend to be long genes, with a minimal gene length of 101 kb. However, many genes longer than 101 kb displayed FGRDs and thus lower coverage of H3K27me3. Therefore, although BGRD genes are long genes, long genes can be associated with either BGRDs or FGRDs.

We then investigated whether it is the gene length or the BGRD that distinguishes oncogenes from other genes. With the BGRD genes being the group *a1* in the gene cluster associated with high H3K27me3 coverage and being longer than 101 kb, we defined the remaining genes longer than 101 kb in the high-coverage cluster as group *a2* (Fig. 2a). Although the repression domains at genes in group *a2* were not yet defined as BGRDs based on our stringent cutoff (121 kb, twofold of the 60.5 kb at the turning point in Supplementary Fig. 1a), most of these repression domains were wider than 60.5 kb and thus were BGRD-like. We further defined the genes that are longer than 101 kb but in the low-coverage cluster as group *b*, and defined the genes shorter than 101 kb as group *c* (Fig. 2a). Groups *a2* and *b* had similar gene lengths (Fig. 2c), whereas repression domains were long in group *a2* but short in group *b* (Fig. 2d), e.g., genes *SYN2* and *WASF3* from group *a2* and genes *PRKCH* and *PRKCQ* from group *b* (Fig. 2e). Both gene length (Fig. 2c) and width of the repression domain (Fig. 2d) were larger in group *a1 *relative to groups *a2* and *b*. We found that group *a1* (long gene and BGRD-associated) and group *a2* (long gene and BGRD-like) were both significantly enriched with oncogenes, whereas groups *b* (long gene and no BGRD) show a slight enrichment, and *c* (short gene) showed little enrichment (Fig. 2f). Meanwhile, oncogenes are overall significantly longer than other genes (Fig. 2g). Therefore, oncogenes are enriched in long genes associated with high coverage of H3K27me3 (BGRD or BGRD-like), but not enriched in long genes marked with low coverage of H3K27me3 (FGRD).

Genes in the group *b* were still slightly enriched with oncogenes (Fig. 2f). GSEA analysis revealed that oncogenes were enriched only in genes with the broadest H3K27me3 in the group *b* (Supplementary Fig. 5a). Group *c* also still showed two clusters that are different in H3K27me3 coverage (Supplementary Fig. 5b). The broadest H3K27me3 in the group *c* was slightly enriched with oncogenes (Fisher’s exact test *p* value 2.1 × 10^−3^, 161 oncogenes observed, 1.3-fold enrichment) (Supplementary Fig. 5c), although not as significant as for the groups *a1* (Fisher’s exact test *p* value 2 × 10^−25^, 111 oncogenes observed, 2.4-fold enrichment) and *a2* (Fisher’s exact test *p* value 1 × 10^−24^, 183 oncogenes observed, twofold enrichment) (Fig. 2f). This result suggested that H3K27me3 width analysis is better at detecting longer oncogenes, whereas it might still capture shorter oncogenes at relatively lower accuracy.

Although oncogenes tend to be long, there were still a few oncogenes that are relatively short. We did a comparison between the shortest 100 oncogenes and 100 neutral genes with similar lengths and expression levels (Supplementary Fig. 5d). H3K27me3 modification was significantly broader on these short oncogenes than on the short neutral genes. Although these short oncogenes are between 0.27 and 13.6 kb long, H3K27me3 signals were strong in a broad region of over 30 kb flanking these oncogenes (Supplementary Fig. 5e), e.g., *MYC* (Supplementary Fig. 5f). BGRDs defined by a less stringent cutoff still showed significant enrichment of oncogenes, although with a less significant *P* value. For example, the 1500 genes covered by 14–28 kb H3K27me3 were still enriched in oncogenes (Fisher’s exact test *p* value 1.8 × 10^−5^, 175 oncogenes observed, 1.4-fold enrichment) (Supplementary Fig. 1c), and the shortest gene in this group is 0.34 kb. Therefore, although we used a very stringent cutoff to define the BGRDs (>121 kb wide), there is space to relax the cutoff in order to capture shorter oncogenes.

We next investigated whether the H3K27me3 width and gene length in CD4^+^ T cell BGRDs are both key to the strong repression of transcription. We first compared two gene sets with similar H3K27me3 widths (Supplementary Fig. 5g) but different gene lengths (Supplementary Fig. 5h), resulting in genes from the longer set having smaller H3K27me3 coverage (Supplementary Fig. 5i). Expression level appeared to be significantly higher for longer genes (Supplementary Fig. 5j). We next compared two gene sets with different H3K27me3 widths (Supplementary Fig. 5k) but similar gene lengths (Supplementary Fig. 5l), resulting in genes with broader H3K27me3 have larger H3K27me3 coverage (Supplementary Fig. 5m), and observed that expression level is significantly lower for genes associated with broader H3K27me3 (Supplementary Fig. 5n). Finally, we compared two gene sets that were different in H3K27me3 width (Supplementary Fig. 5o) and in gene length (Supplementary Fig. 5p) but similar in H3K27me3 coverage (Supplementary Fig. 5q). We observed that expression levels were similar between the two gene groups (Supplementary Fig. 5r). Therefore, H3K27me3 coverage, determined by both H3K27me3 width and gene length, appeared to be the key to the repression of transcription. However, H3K27me3 breadth is better than H3K27me3 coverage in separating oncogenes from other genes, although H3K27me3 coverage also contributes to the separation (Fig. 2h, Supplementary Fig. 5s, and Supplementary Data 3).

Gene length displayed a strong positive correlation with intron length (Supplementary Fig. 5t), but a moderate correlation with exon length (Supplementary Fig. 5u). This is reasonable, because exon length is eightfold shorter than intron length on average. The proportion of intron sequence in a gene increased exponentially upon increase of gene length (Supplementary Fig. 5v). Further, the proportion was significantly larger in oncogenes than in other genes (Supplementary Fig. 5w). Therefore, it is mainly the intron sequences on which the widespread H3K27me3 modification was observed in the repression of transcription at oncogenes.

### BGRD repressed both transcription initiation and elongation

Because BGRDs marked both promoter and gene body, we hypothesized that the promoter and gene body sections of a BGRD may repress different transcriptional stages. Several histone modifications associated with transcription elongation, including H3K36me3 (Supplementary Fig. 6a), H3K79me2 (Supplementary Fig. 6b), and H3K79me3 (Supplementary Fig. 6c), displayed broad depletion in BGRDs. Further, RNA Polymerase II (Pol II) bound the gene body with a lower density for genes in BGRDs (Supplementary Fig. 6d). We calculated Pol II pausing index as the promoter to body ratio of Pol II ChIP-Seq signal^26^ and further calculated the promoter to body (PTB) ratio of H3K27me3. Pol II pausing index negatively correlated with H3K27me3 PTB ratio (Supplementary Fig. 6e). Pol II pausing index was significantly higher for the 10% of genes with the lowest H3K27me3 PTB ratios compared to the 10% of genes with the highest H3K27me3 PTB ratios (Supplementary Fig. 6f). When compared to genes with lower H3K27me3 PTB ratios, H3K27me3 signal appeared higher in the promoter and lower on the gene body for genes with higher H3K27me3 PTB ratios (Supplementary Fig. 6g), whereas Pol II binding signal appeared lower in the promoter and higher on the gene body for genes with higher H3K27me3 PTB ratios (Supplementary Fig. 6h). Therefore, H3K27me3 in the promoter and gene body sections of BGRDs signified repression of transcription initiation and elongation steps, respectively. Notably, H3K27me3 signal in most BGRDs showed enrichment in both promoter and gene body regions (Fig. 1e), whereas Pol II and elongation marks in BGRDs showed depletion in both the promoter and gene body (Supplementary Fig. 6a–d). These results verified that BGRD is associated with repressive effects on both the promoter and gene body, whereas FGRDs is more likely associated with repression of transcription on the promoter only.

### BGRDs were enriched with *cis*-regulatory elements

**Fig. 3:**
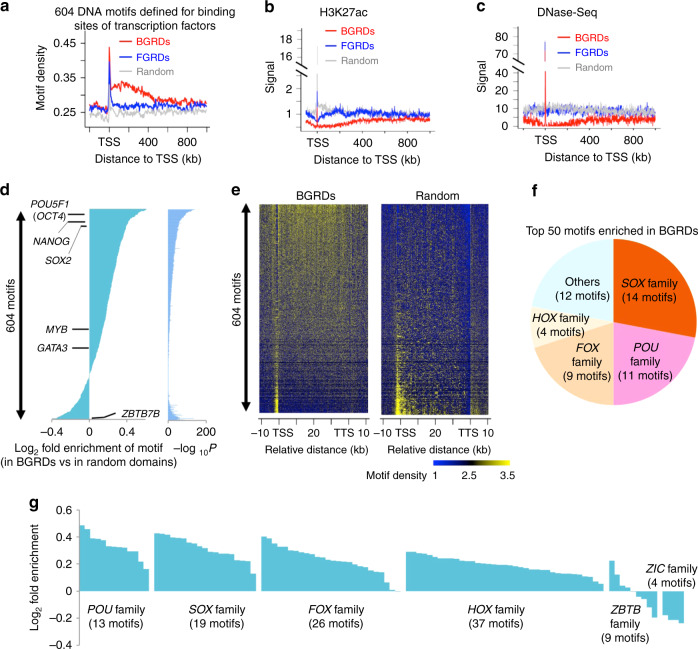
BGRDs in CD4^+^ T cell are enriched with repressed *cis*-regulatory elements. Average density of 604 DNA motifs defined for the binding of individual transcription factors (**a**), average signal of an epigenetic mark for active enhancers (**b**) and chromatin openness defined based on DNase-Seq data (**c**) plotted around TSS associated with each category of repressive domains in CD4^+^ T cell. **d** Fold and *P* value enrichment of individual DNA motifs on genes associated with BGRDs relative to random control genes. Example transcription factors that bind the enriched or depleted DNA motifs were indicated. **e** Heatmaps to show the average density of each motif (row) at each base pair (column) around TSS of genes associated with BGRDs (left) and random domains (right). Motifs were ranked as in **d**. **f** Pie chart to show transcription factor families associated with 50 DNA motifs that are most enriched in BGRD genes relative to random control genes. **g** Fold enrichment of each DNA motif on genes associated with BGRDs relative to random control genes, with the DNA motifs for six transcription factor families presented. *P* values determined by one tail Wilcoxon test **d**. Source data are provided as a Source Data file.

Because H3K27me3 modification could restrict enhancer function^27^, and lack of H3K27me3 on chromatin is essential for the binding of transcription factor to enhancer^28^, we further investigated whether widespread H3K27me3 in BGRDs is associated with the repression of a large cluster of *cis*-regulatory elements. We analyzed 604 DNA motifs defined based on binding sites of transcription factors^29^ and found that these motifs displayed widespread enrichment in BGRDs (Fig. 3a). However, BGRDs displayed broad depletion of active enhancer marker H3K27ac (Fig. 3b) and DNase-Seq signal (Fig. 3c). H3K27me3 density is 2.2-fold higher at BGRD intron motifs than at motifs outside of BGRDs (Supplementary Fig. 7a). There are 120 transcription factors whose binding motifs were enriched in BGRD introns (Supplementary Fig. 7b). Pathways analysis indicated that these transcription factors are significantly enriched in cancer pathways (Supplementary Fig. 7c). Therefore, *cis*-regulatory elements in a silent status displayed a broad enrichment pattern in BGRDs.

However, not all the transcription factors binding motifs show broad enrichment in BGRDs (Fig. 3d). Only the motifs enriched in BGRDs displayed widespread distribution across the gene body (Fig. 3e). The fold enrichment is not very big but statistically significant (Fig. 3d). This is reasonable because these transcription factors may also regulate many genes outside of BGRDs to play functions in normal cell types. The highly enriched motifs belong mostly to 4 transcription factor families, i.e., the *SOX*, *POU*, *FOX,* and *HOX* families (Fig. 3f). The other binding motifs in these four families also displayed slight enrichment in BGRDs (Fig. 3g). We questioned whether the motifs in the CD4^+^ T cell BGRDs could actually be accessible to their associated transcription factors in some other cell types. *OCT4*, *SOX2*, and *NANOG* were known to play key roles in embryonic stem cells, and have 60256, 33555, and 41831 motifs in the BGRD introns of CD4^+^ T cell, respectively (Supplementary Fig. 8a). ChIP-Seq analysis of these factors indicated that these motifs showed enrichment of binding signals of these factors in stem cells (Supplementary Fig. 8b). An important lineage factor for CD4^+^ T cell is the *ZBTB7B* (also known as *ThPOK*)^30^. Among all 604 binding motifs analyzed, the binding motif for *ZBTB7B* was the 4th most depleted (0.76-fold in BGRD relative to random domains) (Fig. 3d).

### BGRDs at oncogenes were conserved across cell types

**Fig. 4:**
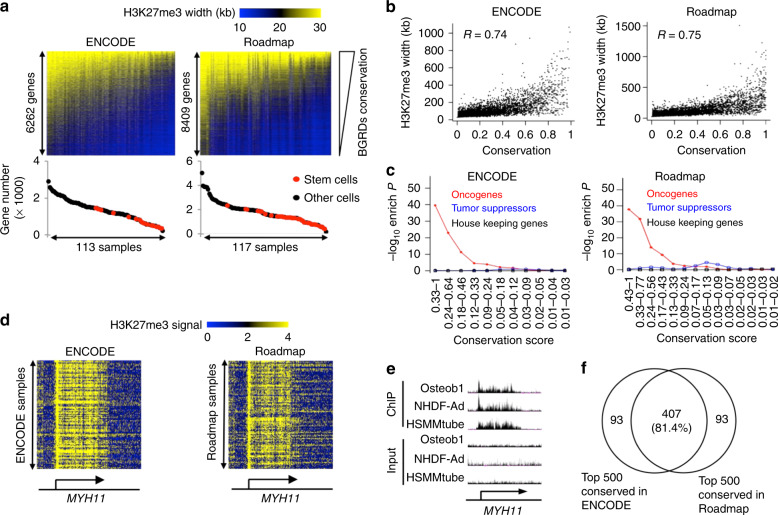
BGRDs at oncogenes are conserved across normal cell and tissue types. **a** Heatmaps to show BGRD width values at 6262 genes (rows) across 113 ENCODE cell samples (columns) (top left panel) and 8409 genes across 117 Roadmap tissue and primary cell samples (top right panel). Presented are genes associated with BGRDs in at least one sample in each heatmap. Genes were ranked by BGRD conservation level defined as the proportion of samples in which the BGRD was observed. Samples were ranked by number of BGRDs. Numbers of genes associated with BGRD in individual samples were plotted below the heatmap. **b** BGRD maximum width plotted against conservation level in ENCODE cell lines (left) and Roadmap tissues and primary cells (right). Pearson correlation coefficient was indicated on top. **c** The enrichment levels of each gene category plotted against conservation level of BGRDs. Genes were ranked by conservation level of BGRDs and divided into groups that each contains 1500 genes, with two neighboring groups in the rank having 500 genes in common. A dot in each curve indicates the enrichment level (*Y*-axis) of one of these groups (range of conservation score indicated on *X*-axis) in the oncogenes, tumor suppressor genes, or housekeeping genes as indicated by the color legends. **d** Heatmaps to show H3K27me3 signal values at individual base pairs (columns) around TSS of the oncogene *MYH11* across ENCODE and Roadmap samples (rows). Arrows indicate gene loci. Gene names were indicated at the bottom. **e** H3K27me3 ChIP signal and matched input background signal at the *MYH11* gene region in three example cell lines. Arrow indicates gene loci. Gene name indicated at the bottom. **f** Venn diagram to show the overlap between genes that are associated with the 500 most conserved BGRDs in the ENCODE samples and in the Roadmap samples. *P* values determined by one tail Fisher’s exact test **c**. Spearman correlation coefficients were indicated **b**. Source data are provided as a Source Data file.

To understand BGRDs beyond the CD4^+^ T cell, we defined 6262 and 8409 genes with BGRDs in at least one of 113 and 117 H3K27me3 ChIP-Seq datasets of non-tumor samples from the ENCODE^31^ and Roadmap Epigenomics Project^32^, respectively (Fig. 4a). We observed a strong correlation between the width of each BGRD and the frequency that BGRDs appeared in these samples (BGRD conservation level) (Fig. 4b). Stem cells tended to have a smaller number of BGRDs but retained the most conserved BGRDs (Fig. 4a). Oncogenes were enriched in the most conserved BGRDs but not in the less conserved groups (Fig. 4c and Supplementary Data 4), e.g., BGRDs highly conserved across ENCODE and Roadmap datasets were observed at the oncogene *MYH11*^33^ (Fig. 4d, e). The majority (81.4%) of the 500 most conserved BGRDs were shared between the ENCODE and Roadmap samples (Fig. 4f).

### Widespread alternation of BGRDs in cancer cells

**Fig. 5:**
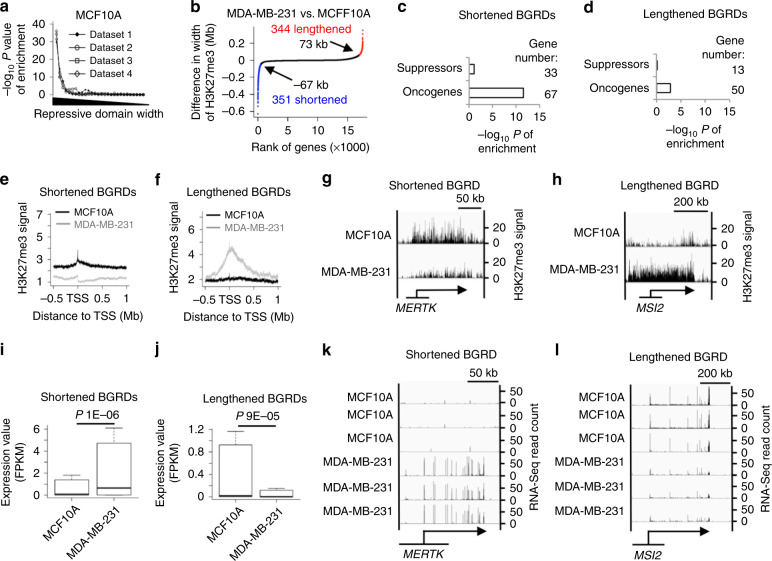
Disruption of BGRD is associated with upregulation of oncogenes in breast cancer cells. **a** Enrichment level of oncogenes plotted against width of repression domain in breast epithelial cell lines. Genes were ranked by H3K27me3 width and divided into groups that each contains 1500 genes, with two neighboring groups in the rank having 500 genes in common. A dot in each curve indicates the enrichment level (*Y*-axis) of one of these groups (*X*-axis) in the oncogenes. **b** Cumulative plot of difference in H3K27me3 width between the cells MDA-MB-231 and MCF10A. Genes were ranked by difference in H3K27me3 width from the most shortened (blue) at the left side of *X*-axis to the most lengthened (red) at the right side. Enrichment level and number of oncogenes or tumor suppressors in genes that show shortening (**c**) or lengthening (**d**) of BGRDs in MDA-MB-231 relative to MCF10A cells. Average ChIP-Seq signal of H3K27me3 around TSS of genes that show shortening (**e**) or lengthening (**f**) of BGRDs in MDA-MB-231 relative to MCF10A. **g**, **h** ChIP-Seq signal of H3K27me3 at example genes in the MDA-MB-231 and MCF10A cell lines. Arrows indicate gene loci. Gene names were indicated at the bottom. *Y*-axis scale at the right side indicates ChIP-Seq signal strength. Boxplot for expression values of genes that show shortening (**i**) or lengthening (**j**) of BGRDs in MDA-MB-231 relative to MCF10A, *n*(lengthened) = 344 and *n*(shortened) = 351. Box plots: center line is median, boxes show first and third quartiles, whiskers extend to the most extreme data points that are no more than 1.5-fold of the interquartile range from the box. RNA-Seq signal at example genes that display shortening (**k**) or lengthening (**l**) of BGRDs in MDA-MB-231 relative to MCF10A cells. Arrows indicate gene loci. Gene names were indicated at the bottom. *Y*-axis scale at the right side indicates RNA-Seq signal. *P* values determined by one tail Fisher’s exact test **a**, **c**, **d** or one tail Wilcoxon test **i**, **j**. Source data are provided as a Source Data file.

Because tumor development can be caused by upregulation of oncogene expression, we hypothesized that BGRDs at some oncogenes might be disrupted in cancer cells. We first analyzed ChIP-Seq datasets for H3K27me3 in non-tumor breast cell line MCF10A (Fig. 5a and Supplementary Data 5a), prostate epithelial (PrEC) cell (Supplementary Fig. 9a and Supplementary Data 5b), CD4^+^ T cell (Supplementary Fig. 10a, Supplementary Data 1a), primary mammary epithelial (HMEC) cell (Supplementary Fig. 12a and Supplementary Data 5c), and human epidermal melanocytes (HEM) cell (Supplementary Fig. 14a and Supplementary Data 5d). BGRDs in these cells were enriched at oncogenes too. The cumulative plot of the increased (or decreased) widths of individual BGRDs in the breast cancer cell MDA-MB-231 relative to the MCF10A appeared to be close to an L shape (Fig. 5b), with the turning points appeared at ~35 kb. To define a shortened or lengthened BGRD, we required the decreased or increased width of H3K27me3 to be twofold of the value at the turning points (Fig. 5b). This allowed us to define 351 and 344 genes associated with shortened and lengthened BGRDs, respectively, in the MDA-MB-231 relative to the MCF10A. In a similar approach, we found 380 and 353 genes associated with shortened and lengthened BGRDs, respectively, in the prostate cancer cell LNCaP relative to the PrEC (Supplementary Fig. 9b). For fair comparison between these gene groups, we decided to use the top 350 genes associated with each type of change in BGRD width for further analyses.

Intriguingly, we found that not only the shortening, but also the lengthening of BGRDs were enriched at oncogenes but not at tumor suppressors (Fig. 5c, d and Supplementary Fig. 9c, d). However, the enrichment at oncogenes was stronger for the shortening of BGRDs. A change in the breadth of the repressive domain was accompanied by a change in H3K27me3 signal density (Fig. 5e–h and Supplementary Fig. 9e–h). Notably, for *MERTK*^34^, a well-known oncogene for breast cancer, we observed disruption of BGRD in MDA-MB-231 relative to MCF10A (Fig. 5g). Further, *TMPRSS2*^35^, a well-known oncogene in prostate cancers, displayed disruption of BGRD in LNCaP relative to PrEC (Supplementary Fig. 9g). The shortening and lengthening of BGRDs correlated with up- and downregulation of RNA expression, respectively (Fig. 5i–l and Supplementary Fig. 9i–l). We performed several additional comparisons between cancer cells and matched non-cancer cells, including the leukemia cell Jurkat versus CD4^+^ T cell, breast cancer cell line MCF7 versus MCF10A, MDA-MB-231 versus HMEC, MCF7 versus HMEC, and melanoma cell SK-MEL-28 versus HEM (Supplementary Figs. 10–14). In each of these comparisons, we consistently observed that shortening of broad H3K27me3 was significantly associated with oncogene upregulation, whereas the lengthening of BGRDs was less often associated with known oncogenes in cancer cell lines.

**Fig. 6:**
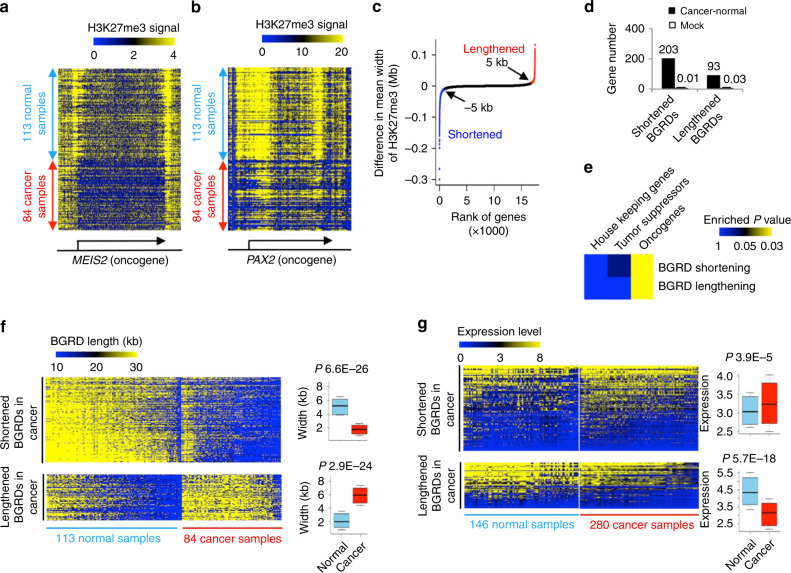
Systematic pan-cancer analysis revealed widespread shortening of BGRDs at oncogenes. Heatmap to show H3K27me3 ChIP-Seq signal at individual base pairs (columns) around TSS of the oncogene *MEIS2* (**a**) and *PAX2* (**b**) in individual samples (rows). Arrows indicate gene loci. Gene names were indicated at the bottom. **c** Cumulative plot of difference in average H3K27me3 width between normal and cancer samples. Genes were ranked by difference in H3K27me3 width from the most shortened at the left side (blue) of *X*-axis to the most lengthened (red) at the right side. **d** Number of genes that displayed shortening or lengthening of BGRDs in 84 cancer cell samples relative to 113 non-cancer cell samples. Mock analysis was performed after shuffling sample labels to randomly assign 84 samples as mock cancer samples. Data are presented as mean ± SD. **e** Heatmap to show one tail Fisher’s exact test *P* value for the significance of overlap between oncogenes, tumor suppressors, or housekeeping genes and genes that display lengthening or shortening of BGRDs in 84 cancer cell samples relative to 113 non-cancer cell samples. Heatmap to show BGRD widths (**f**) and expression values (FPKM) (**g**) of individual genes (row) in individual samples (columns), with box plots to further indicate difference in BGRD widths or expression values between normal and cancer samples, n(shortened) = 203 and n(lengthened) = 93. Box plots: center line is median, boxes show first and third quartiles, whiskers extend to the most extreme data points that are no more than 1.5-fold of the interquartile range from the box. *P* values determined by one tail Fisher’s exact test **e** or one tail Wilcoxon test **f**, **g**. Source data are provided as a Source Data file.

Although different patients might be caused by different oncogenes, the same oncogene can cause different types of cancer. Therefore, we performed a pan-cancer analysis between the 84 H3K27me3 ChIP-Seq data from ENCODE cancer cell lines^31^ and the 113 samples from ENCODE non-cancer cell lines. We detected 8105 genes with BGRDs in at least one of these samples. Many oncogenes, such as *MEIS2*^36^ (Fig. 6a) and *PAX2*^37^ (Fig. 6b), had conserved BGRDs across normal cells but displayed BGRD shortening in cancer cells. The cumulative plot of the increased (or decreased) widths of individual BGRDs appeared close to an L shape, with the turning point appearing at ~5 kb (Fig. 6c). By requiring a difference in BGRD mean width to be larger than 5 kb and the difference in BGRD conservation level to be larger than 0.3 (normal distribution *P* value 0.01), we defined 203 genes with BGRD shortening in cancers, which is 2.2-fold more than the 93 genes with BGRD lengthening (Fig. 6d), whereas only 0.03 and 0.01 genes with BGRD shortening and lengthening were observed in mocked comparison, respectively (Fig. 6d). Lengthening and shortening of BGRDs were both enriched in oncogenes but not in tumor suppressors (Fig. 6e). We next collected 280 and 146 mRNA-Seq datasets from the ENCODE cancer and normal cells^31^, respectively. Lengthening and shortening of BGRDs appeared to be associated with repression and activation of transcription, respectively (Fig. 6f, g). We also observed similar results using an independent set of microarray expression data from the ENCODE project^31^ (Supplementary Fig. 15a). These results were also reproducible when we required that the ChIP-Seq and mRNA-Seq data be obtained from the same set of cell lines (Supplementary Fig. 15b, c). Therefore, widespread reprogramming of BGRD length is associated with dysregulation of oncogene expression in cancer cells.

### BGRD analysis for discovery of tumor-promoting genes

**Fig. 7:**
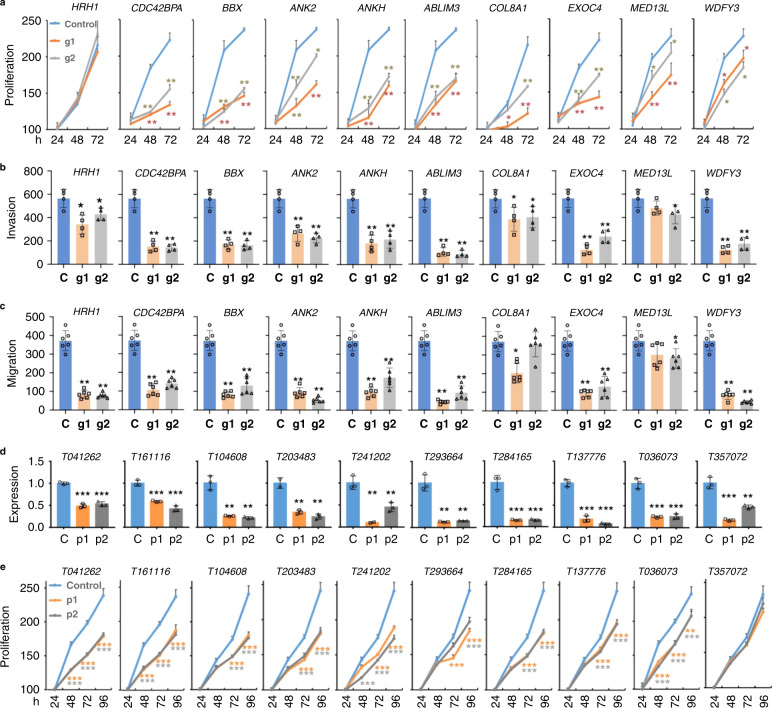
Depletion of putative tumor-promoting genes and lncRNAs identified using BGRD signature impaired cancer phenotypes of tumor cells. Proliferation (**a**), transwell invasion (**b**), and migration (**c**) of breast cancer cell MDA-MB-231 with each putative tumor-promoting gene disrupted by CRISPR-Cas9 guide RNA g1, g2, or under control condition. LncRNA expression level (**d**) and cell proliferation rate of breast cancer cell MDA-MB-231 (**e**) with each candidate tumor-promoting lncRNA disrupted by CRISPR-Cas9 guide RNA pairs p1, p2, or under control condition. All the data were analyzed from at least three independent experiments and the quantification results were expressed as mean ± SD. *P* values determined by two tails Student’s *t* test and the exact *P* values were listed in the Source data; ***P < 0.001; **P < 0.01; *P < 0.05; For proliferation, *n* = 5; for migration, *n* = 6; for invasion, *n* = 4; for lncRNA expression level, *n* = 3. C: control; KO: knockout; g1: guide RNA #1; g2: guide RNA #2; P1: guide RNA pair #1; P2: guide RNA pair #2. Source data are provided as a Source Data file.

We collected a total of 735 existing ChIP-Seq datasets for H3K27me3 in the GEO and ENCODE databases, and then employed a forward feature construction method that identified 36 of these datasets as the most useful combination in recapturing the reported oncogenes by BGRDs (See “Methods”) (Supplementary Fig. 16a). We next developed a pipeline to define putative tumor-promoting genes not defined by previous mutation analysis, expressed in a given cancer cell type, and each with an BGRD length decreased by at least 50% in the given cancer cell type relative to mean length in the 36 datasets (Supplementary Fig. 16b). These analyses resulted in 420 and 232 candidate tumor-promoting genes for triple negative breast cancer cell MDA-MB-231 and prostate cancer cell LNCaP, respectively. For experimental verification, we randomly selected ten candidates for the MDA-MB-231 cell, then manually inspected the H3K27me3 signal pattern to confirm BGRD shortening at each candidate, and searched literature to confirm that the candidate had not been previously reported as an oncogene. The candidates that did not pass the confirmation were replaced with new randomly selected candidates until all candidates passed the confirmation. We then disrupted each candidate gene using two CRISPR-Cas9 guide RNAs whose cutting efficiencies were confirmed by T7 Endonuclease I Assay (Supplementary Fig. 16c). We further performed western blot to verify efficiency of gene disruption at the protein level for *CDC42BPA*, *ANK2*, *EXOC4,* and *BBX*. We found that the protein level of the four genes was significantly down regulated by either of two CRISPR gRNAs (Supplementary Fig. 16d–g). We observed a significant decrease in cell proliferation (Fig. 7a), invasion (Fig. 7b), and migration (Fig. 7c) for nine, nine, and eight genes, respectively. We also tested ten candidates defined in this approach for prostate cancer cell LNCaP and observed a significant decrease in cell proliferation for nine genes when deleted by CRISPR-Cas9 (Supplementary Fig. 16h, i). For comparison, we next tested ten genes identified in the same approach except that their BGRD was lengthened in cancer cells (Supplementary Fig. 17a, b). We observed a significant decrease in cell proliferation for only one of these ten genes when disrupted by CRISPR-Cas9 (Supplementary Fig. 17c, d). In addition to the T7 Endonuclease I Assay to verify disruption of these ten genes, we performed western blot for *RALGPS1* and *VSNL1*, the two control genes that have the lowest RNA expression among these ten control genes. The result indicated that these two genes both have protein expression in the MDA-MB-231 cell, whereas their protein expression was down regulated by the CRISPR-Cas9 experiment (Supplementary Fig. 17e). We further observed BGRDs at some reported tumor-promoting lncRNAs, e.g., the *HOTAIR*^38^ and *SCHLAP1*^39^ in MCF10A cell line (Supplementary Fig. 18a). We next defined 213 putative tumor-promoting lncRNAs expressed in MDA-MB-231 and showed shortened BGRDs in MDA-MB-231 relative to the 36 datasets (Supplementary Fig. 18b). We then experimentally tested ten candidates that were further manually confirmed to show BGRD shortening and were not reported in literature to be tumor-promoting. The knockouts for eight of these ten candidates significantly impaired the proliferation of MDA-MB-231 (Fig. 7d, e and Supplementary Fig. 18c). These results suggest that the BGRD is a useful signature for mutation-independent discovery of epigenetically altered tumor-promoting factors.

## Discussion

After we observed that the shortening of BGRDs in cancer cells relative to non-cancer cells is associated with oncogenes, we reasoned that BGRDs lengthening in cancer samples might be enriched at tumor suppressor genes. However, the results indicate that BGRD lengthening is not significantly enriched at tumor suppressor genes. Therefore, cancer cells might need repression of some tumor suppressors, but do not develop BGRD for the repression. Notably, there were oncogenes repressed by lengthened H3K27me3 in cancer cells relative to normal cells. It suggested that it is the BGRD rather than the shortening or lengthening of BGRD that is associated with oncogenes. Instead, the shortening and lengthening of BGRD would be associated with up and down regulation of these genes, respectively.

Although the oncogenes that cause a cancer tend to be upregulated in the cancer, discovering these oncogenes solely by expression analysis is still challenging. In differential analysis between MDA-MB-231 and MCF10A, or between LNCaP and PrEC, the 1500 most upregulated genes show little enrichment in oncogenes (Supplementary Fig. 19a, b and Supplementary Data 6a, b). In contrast, the 1500 most shortened H3K27me3 domains show significant enrichment in the oncogenes (Supplementary Fig. 19c, d and Supplementary Data 6c, d). Therefore, BGRD shortening can be a better signature for oncogene discovery when compared to expression upregulation, probably because many other genes can also be among the most upregulated genes in cancer but are not among the most shortened H3K27me3. Notably, due to technical limitation on the accuracy of BGRD width calculation based on current ChIP-Seq technique, further manual inspection to ensure shortening of BGRDs is recommended.

A broad H3K27me3 that is conserved across normal cell types may be used to define pan-cancer oncogenes, whereas the shortening of broad H3K27me3 in individual cancer samples may characterize patient-specific oncogene dysregulation. For example, although 153 genes showed shortening of broad H3K27me3 in both MDA-MB-231 and LNCaP, there were also 267 and 79 genes that showed the shortening only in MDA-MB-231 cell and only in LNCaP cell, respectively (Supplementary Fig. 20a), e.g., the *EGFR*^40–42^ in both MDA-MB-231 and LNCaP, and the *TMPRSS2*^35,43^ in only LNCaP (Supplementary Fig. 20b).

H3K27me3 domains marking entire genes, as well as forming broad domains encompassing many genes on the Mb-scale have been reported in *D. melanogaster*^44,45^, *C. elegans*^46^, and mESCs^1^. These include Broad H3K27me3^1^, LOCK^25^ and BLOC^47^. Although BGRD and these previously reported types of broad repression domains are all broad, the difference in the methods to define them determined that they are still quite different in definition and in biological implication. It is also possible that the heterogeneity of broad repression domains is not yet fully covered by these reported types. Using the exact terminology for each type of these broad repressive domains and using the right bioinformatics method for the detection of each type will be important to accurately understand the biological implication of these domains.

## Methods

### Source of datasets analyzed in this project

The accession numbers for each piece of raw data from public database or the source of processed dataset, along with the IDs of figures in which each dataset was analyzed, were indicated in the Supplementary Data 7. Human reference genome sequence version hg19 was downloaded from the UCSC Genome Browser website (https://genome.ucsc.edu)^48^. The reference gene list is downloaded from the website of the bioinformatics tool GREAT (http://great.stanford.edu/public/html/)^49^. Overall, 1500 oncogenes and 1500 tumor suppressor genes were collected from a recent publication^21^. Overall, 330 genes in the Pathways In Cancer (hsa05200) were collected from the Kyoto Encyclopedia of Genes and Genomes (KEGG) database (https://www.genome.jp/kegg/)^50–52^. Overall, 3804 housekeeping genes were collected from a recent publication^24^ (see Supplementary Data 8).

### General bioinformatics analysis

For all box plots in this manuscript, the bottom, middle and top lines indicate the first, second and third quartiles, respectively, with the whisker range defined as 0.2. We used one tail Mann–Whitney–Wilcoxon test to determine the significance of difference in boxplot between groups, calculated by the R function wilcox.test(). Fisher’s exact test was performed using a python script developed in this project and available at the website FigShare (fisher.test.enriched.py, 7676087). KEGG pathway analyses were performed by DAVID database version 6.8 (https://david.ncifcrf.gov)^53,54^.

### Bioinformatics analysis of RNA-Seq and microarray data

RNA-Seq raw reads were mapped to the human genome version hg19 using TopHat version 2.1.1^55^ with default parameter values. Expression value (FPKM) for each gene was determined by the function Cuffdiff in Cufflinks version 2.2.1^55^ with default parameter values. The software MeV version 4.8.1^56^ was used to draw the heatmaps for gene expression analysis. We utilized the function genomeCoverageBed in BEDTools Version 2.16.2^57^, along with the nor2total function in DANPOS version 2.2.2^58,59^, and the tool bedGraphToBigWig (https://www.encodeproject.org/software/bedgraphtobigwig/)^60^ to generate a BigWig file that contains RNA-Seq signal (read density) at each base pair across the genome. The BigWig file was then submitted to the UCSC Genome Browser to visualize RNA-Seq signal at individual genes. We downloaded Affymetrix array data from the ENCODE project website and Affymetrix U133 Plus 2.0 array data for CD4^+^ T cells from the Gene Expression Omnibus (GEO; accession codes available in Supplementary Data 7). We then used the ReadAffy and RMA functions in the Affy R package to calculate the expression value for each gene.

### Bioinformatics analysis of ChIP-Seq and DNA methylation

ChIP-Seq datasets downloaded from the National Heart, Lung, and Blood Institute (NHLBI) website (https://dir.nhlbi.nih.gov/papers/lmi/epigenomes/hgtcell.aspx)^61^ are the sequencing reads in BED format, which contain the genomic location of each read that has been mapped to the human genome version hg18. We converted these data from the hg18 to the hg19 version of the human genome using the tool LiftOver downloaded from the UCSC Genome Browser website (http://hgdownload.cse.ucsc.edu/downloads.html)^48,60^. For other ChIP-Seq, MRE-Seq and MeDIP-Seq data downloaded as sequencing reads in FASTQ format from the GEO or ENCODE databases, the reads were mapped to the human reference genome version hg19 using bowtie version 1.1.0^62^ with default parameter values.

We then submitted the mapped reads to the Dregion function in DANPOS version 2.2.2 (https://sites.google.com/site/danposdoc/)^58,59^ to calculate ChIP-Seq signal (read density) at each base pair of the genome, subtract background input signal, normalize read number, and define individual enrichment peaks. The Dregion stored the signal value at each base pair in a Wiggle format file, which we next converted to BigWig format using the tool WigToBigWig (https://www.encodeproject.org/software/wigtobigwig/). The BigWig files were submitted to the UCSC Genome Browser (https://genome.ucsc.edu) to visualize ChIP-Seq signal at each base pair^48,63^. The Dregion also stored individual feature values for each enrichment peak of ChIP-Seq signal. These feature values include peak width and height. To calculate signal value at each base pair across each gene, we used the Profile function in DANPOS version 2.2.2^58,59^. The ChIP-Seq signal values at individual base pairs across individual genes were then submitted to the software MeV version 4.8.1^56^ to draw heatmaps. The Profile function in DANPOS 2.2.2^58,59^ was also used to calculate average ChIP-Seq, MRE-Seq, or MeDIP-Seq signal at each gene group.

### Method to define BGRDs and FGRDs and their associated genes

After peak calling for H3K27me3 ChIP-Seq dataset from the CD4^+^ T cell, we employed the Selector function in DANPOS version 2.2.2^58,59^ to retrieve the enrichment peaks that overlapped with each gene. A Perl script was developed to calculate the total width of all peaks mapped to each gene (region.sum.perl, 7676087). Also, a Perl script was developed to calculate the maximal height of all peaks mapped to each gene (peak.max.perl, 7676087). We then plotted the maximal height of H3K27me3 peaks against the total width of H3K27me3 peaks for each gene, and observed that the subset of genes associated with the largest height value for H3K27me3 is different from the subset of genes that have the largest width value for H3K27me3 (Fig. 1d). We found that the cumulative plot of H3K27me3 width is close to an L shape. Because we used the 17,533 genes that were well annotated by the developer of the bioinformatics tool GREAT, we normalized the *x*-axis and *y*-axis in the figures (Figs. 5b, 6c, and Supplementary Figs. 1a, 9b, 10b, 11b, 12b, 13b, 14b) both to have a scale range between 0 and 17,532. We then calculated the Euclidean distance from each gene in the curve to the right bottom point of the figure (*x* = 17,532, *y* = 0). The gene with the smallest Euclidean distance will be the turning point. So the H3K27me3 width appears to be 60.5 kb at the turning point of the L shape (Supplementary Fig. 1a). We therefore used an H3K27me3 width at 121 kb (twofold of the width at the turning point) as the cutoff and define 500 BGRDs. For a fair comparison with BGRDs, we defined 500 FGRDs as these that displayed the largest height value of H3K27me3 enrichment peaks. For consistency across the manuscript and fair comparison between datasets, we thereafter defined BGRDs as the top 500 that displayed the most widespread H3K27me3 and FGRDs as the top 500 that displayed the highest H3K27me3 peaks in each other dataset.

### Method to define genes associated with LOCKs

We collected LOCKs for human placenta and mouse brain in a recent publication^25^. For human placenta, we converted LOCKs from the human genome version hg18 to the version hg19 using the tool LiftOver (https://genome.ucsc.edu/util.html). We used the Selector function in DANPOS version 2.2.2 to retrieve genes overlapping with LOCKs. For mouse brain, we converted LOCKs from the mouse genome version mm8 to the version mm9 using the tool LiftOver. The MGI database (http://www.informatics.jax.org/homology.shtml) was employed to identify homologous genes between mouse and human. The mouse homologs of human oncogenes were recognized as mouse oncogenes to analyze their overlap with mouse LOCK-associated genes and mouse BGRD-associated genes.

### Analysis of transcription factor binding motifs

To analyze the density of transcription factor binding motifs across the genome, we developed a Python script (towig.py, 7683872) to generate a Wiggle format file for the motifs. In this Wiggle format file, each base pair that overlapped with at least one motif was assigned a value 1, whereas a base pair that did not overlap with any motif was assigned a value 0. The Wiggle file was submitted to the Profile function of DANPOS version 2.2.2^58,59^ to plot average density of motifs around TSS in each gene group. To define enrichment level of a motif in the gene group associated with BGRDs relative to the gene group associated with random domains, we also generated a Wiggle format file using the given motif. Further, different lengths of regions have been taken care of by scaling the gene body to the same length. Average density of the motif on genes was calculated for each gene group, and then a fold difference in the density was calculated between the gene group associated with BGRDs and the gene group associated with random domains.

### Pol II pausing index and H3K27me3 PTB ratio

We developed a Python script that is available at our project website (ptb.script.r, 7676099). The Pol II pausing index was calculated as the promoter (defined as transcript start site upstream 30 bp to downstream 300 bp) to body (defined as transcript start site downstream 300 bp to transcript terminate site) ratio of Pol II ChIP-Seq signal (the sum of ChIP-Seq read density at each base pair within a given region, e.g., a promoter region), as was described before^59^. Similarly, we calculated an H3K27me3 PTB ratio for each gene. To take the broader distribution of H3K27me3 around TSS into account, we defined promoter regions as from transcript start site upstream 3 kb to downstream 3 kb, and defined gene body region as from transcript start site downstream 3 kb to transcript terminate site, respectively. To examine the relationship between Pol II pausing index and H3K27me3 PTB ratio (Supplementary Fig. 6e), we restricted the gene set to only include the ones showing H3K27me3 peaks and detectable Pol II density (with the average density of Pol II at both promoter and gene body larger than 0.01). To quantitatively compare genes with high and low H3K27me3 PTB ratios (Supplementary Fig. 6f), we selected the top, median and bottom 1000 genes according to their H3K27me3 PTB ratios.

### Determine 36 optimal datasets for recapturing oncogenes

A collection of 735 ChIP-Seq datasets for H3K27me3 were downloaded from public databases (GEO and ENCODE), with the accession numbers available in Supplementary Data 9. For each dataset, we defined genes that are associated with BGRDs, and calculate a *P* value based on Fisher’s exact test to assess their significance of overlap with oncogenes. We ranked samples by the *P* values from the smallest to the largest. We then combined the genes from each top-ranked sample to form a combined nonredundant gene list, and calculated *P* value for the overlap between the combined gene list and oncogenes. At the beginning, the *P* value decreased along with the increase of sample number. Thereafter, the *P* value starts to increase. We finally determined to use 36 top-ranked optimal samples, as the *P* values for larger number of samples become larger than that for using only the single top-ranked sample (Supplementary Fig. 16a and Supplementary Table 1).

### BGRD shortening or lengthening analysis

For fair comparison across samples, H3K27me3 widths at individual genes in all samples were normalized to the widths in CD4^+^ T cell using Quantile normalization method. Since the cumulative plot of decreased (or increased) widths of BGRDs is close to an L shape, our cutoffs to define shortened (or lengthened) BGRDs were based on the width value at the turning point of the L shape. Since the conservation level of BGRD is a proportion value that should follow a normal distribution, the increase or decrease of conservation value would also follow a normal distribution. Lengthened or shortened BGRDs in ENCODE cancer cell lines relative to ENCODE non-cancer cell lines were defined by requiring a difference in mean width of H3K27me3 to be larger than 5 kb (near the turning point of the L shape in Fig. 6c), and by further requiring the difference in BGRD conservation level to be larger than 0.3 (normal distribution *P* value 0.01). As a control analysis to define the number of lengthened or shortened BGRDs expected by chance, we randomly shuffled the labels for ChIP-Seq samples to generate mocked normal and cancer labels for the cell lines, and repeated the comparison for 1000 times based on mocked sample labels. To define shortened or lengthened BGRDs in a single cancer type, we required the difference in H3K27me3 width to be twofold of the width value at the turning point of the L-shape cumulative plot. To compare H3K27me3 width between the 36 optimal samples and any given cancer cell (Supplementary Fig. 16b), we used genes that displayed BGRDs in at least two of these 36 samples. This resulted in 1446 unique genes that we defined as putative oncogenes. To limit the putative oncogenes to those that have failed to be detected by cancer genome analysis, we excluded the ones that possibly have an oncogenic or tumor suppressive genetic alternation, with an aggressive *Q* value cutoff 0.5 calculated by TUSON^21^. We next selected putative oncogenes that are likely to play a role in a cancer cell line by requiring their expression level to be higher than median level of all genes and further by requiring H3K27me3 width to be at least 50% shorter than the mean width in the 36 top-ranked samples. This resulted in 420 and 232 candidates for the MDA-MB-231 and the LNCaP cells, respectively (Supplementary Fig. 16b). To define the 164 genes with H3K27me3 lengthened in the MDA-MB-231 cell, except requiring H3K27me3 to be at least 50% broader than the mean width in the 36 optimal samples, all other requirements are the same with the requirements described above for the genes with H3K27me3 shortened in cancer cells (Supplementary Fig. 17a). To define putative oncogenic lncRNAs, the reference lncRNA list is downloaded from the website MiTranscriptome^64^. From the 1810 lncRNAs with BGRDs observed in at least two of the 36 optimal samples, we narrowed down the list for MDA-MB-231 cell by requiring their expression level to be higher than median level of all genes, and further requiring that H3K27me3 width is at least 50% and at least 20 kb shorter than the mean width in the 36 optimal samples. This resulted in 213 candidates for the MDA-MB-231 (Supplementary Fig. 18b).

### Bioinformatics methods related to oncogenes verification

We randomly selected ten candidate genes with BGRD shortening in MDA-MB-231 and ten candidate genes with H3K27me3 shortening in LNCaP. We then further manually inspected their H3K27me3 pattern to confirm BGRD shortening. Briefly, we required their H3K27me3 to be widespread in multiple normal cell lines but clearly depleted in MDA-MB-231 or LNCaP. To determine the promoters of candidate lncRNAs for CRISPR-Cas9 cutting, we further manually confirmed that the promoter regions showed the promoter epigenetic mark H3K4me3 in multiple cell types. We further selected ten candidate genes with H3K27me3 lengthening in MDA-MB-231, and manually inspected the H3K27me3 pattern to confirm that their H3K27me3 was depleted in multiple normal cell types but clearly broader in MDA-MB-231. We also further searched literature to confirm that these candidates were not reported to be oncogenes in existing publications. When a randomly selected candidate did not pass the manual confirmations, we replaced it with another randomly selected candidate that was then also submitted to manual confirmation. We repeated this procedure until all ten randomly selected candidates in each category have passed the manual inspections.

### CRISPR gRNA and lentiviral vector design

Two open-access software programs, Cas-Designer (http://www.rgenome.net/cas-designer/) and CRISPR design (http://crispr.mit.edu/), were used to design guide RNAs (gRNA) targeted to candidate gene. Two guides were designed for each gene. For each tumor-promoting lncRNA candidate, two pairs of gRNAs were designed to delete 5-5.5 kb of the promoter region defined as from 5 kb upstream to 1 kb downstream of TSS^65^ (gRNA sequence were listed in Supplementary Data 10). Target DNA oligos were purchased from IDT (Integrated DNA Technologies). The pair of oligonucleotides for each target were annealed by heating to 95 °C for 5 min and cooling to 85 °C at 2 °C per second and then further down to 25 °C at 0.1 °C per second. The annealed oligos were cloned into the lentiCRISPR v2 plasmid (Addgene plasmid# 52961) via BsmBI restriction enzyme sites upstream of the scaffold sequence of the U6-driven gRNA cassette. All plasmids were sequenced to confirm successful ligation.

### Lentiviral constructs

Lentivirus was packaged by co-transfection of constructs with second-generation packaging plasmids pMD2.G, psPAX2 (Addgene plasmids #12259 and #12260) into six-well plate with HEK293T cells. For each virus, HEK293T cells were transfected with 250 ng of pMD2.G, 750 ng of psPAX2, 1 *μ*g of target plasmid diluted in Opti-MEM. After the first 24 h of transfection, the medium was changed to fresh DMEM with 10% FBS, and the viral supernatants of 48 and 72 h after transfection were pooled and centrifuged at 800 g for 10 min to pellet cell debris. Then, the supernatant was filtered through a 0.45-µm filter, and used immediately for infection.

### Cell culture and lentiviral transduction

MDA-MB-231 and LNCaP were purchased from ATCC. All cells used in this study were within 20 passages after receipt. MDA-MB-231 was maintained in vitro in DMEM medium supplemented with 10% FBS, 2 mmol per l-glutamines. LNCaP was cultured in 5% CO_2_ and maintained in vitro in RPMI 1640 medium supplemented with 10% FBS, 2 mmol per l-glutamines. Those cell lines were mycoplasma negative during routine tests. Cells were grown to 70% confluence and infected with lentivirus containing 10 µg per mL polybrene. The media was changed 16 h after viral transduction and infected cells were incubated for 48 h before selection with 1.5 μg per mL puromycin for 3 days. Cells were collected then for cell proliferation assay, transwell invasion assay, migration assay, and total RNA and genomic DNA extraction.

### T7 endonuclease I assay of CRISPR/Cas9 induced mutation

Genomic DNA from cells transduced with lentivirus was extracted with a Quick-DNA Miniprep Kit (Zymo Research, Irvine, CA) following manufacturer’s protocol, and quantified using a Synergy 2 Multi-Mode Reader (BioTek, Winooski, VT, USA). The targeted regions were PCR-amplified with amfiSure PCR Master Mix (GenDEPOT, Barker, TX, USA) using primers flanking the target sites (primers were listed in Supplementary Data 11). Overall, 200 ng of PCR products were denatured and then slowly hybridized to form heteroduplexes using the following program settings: 95 °C for 5 min, 95–85 °C at −2 °C per second, 85–25 °C at −0.1 °C per second. Heteroduplexes were digested with T7 endonuclease I (New England Biolabs, Ipswich, MA, USA) at 37 °C for 30 min. And the digested products were separated on a 2% TAE agarose gel for analysis. Images were captured using the ChemiDoc XRS+ Molecular Imager system (BIO-RAD, Hercules, CA, USA).

### Verification of CRISPR/Cas9 induced lncRNA deletion

To verify the mutagenesis on tumor-promoting lncRNA candidates, PCR was performed to amplify the targeted region on promoters. In wild type cells, PCR primers flanking the deleted regions will generate an ~6 kb fragment. However, the same primers will give us shorter fragments in the edited cells because of the mutation caused by gRNA pairs. The RNA levels of those lncRNA candidates were also detected after CRISPR/Cas9 editing. RNA of edited cells was extracted using Quick-RNA Mini-prep (Zymo Research, Irvine, CA), and cDNA was obtained using amifiRivert cDNA Synthesis Platinum Master Mix (GenDEPOT, Barker, TX, USA). Real-time PCR was performed with SYBR Green Master Mix (BIO-RAD, Hercules, CA, USA). The primers used for PCR and RT-PCR were listed in Supplementary Data 12.

### Cell proliferation assay

Cells after viral transduction and puromycin selection were plated in 96-well plates at a density of 4000 cells per well for MDA-MB-231 and allowed to attach for 24 h. Viability was measured utilizing the CellTiter-Glo® Luminescent Cell Viability Assay (Promega, Madison, WI, USA). Results were read at 24, 48, and 72 h on the Synergy 2 Multi-Mode Reader. Cell proliferation method of LNCaP is similar to MDA-MB-231, except that LNCaP was cultured in 5% CO_2_ and maintained in vitro in RPMI 1640 medium supplemented with 10% FBS, 2 mmol per l-glutamines.

### Migration and invasion assays

Cell migration assays were performed using Transwell chambers (8 μM pore size). A total of 5 × 10^4^ MDA-MB-231 cells (300 µl) after viral transduction in DMEM medium with 0.5% FBS were added to the upper chamber. In total, 800 µl DMEM medium with 0.5% FBS were added to the bottom wells of the chambers. After 4 h, the cells that had not migrated were removed from the upper face of the filters using cotton swabs, and the lower surfaces of the filters were fixed with methanol and stained with 0.5% Crystal Violet Staining Solution. The migratory cells were counted in three random chosen areas from each membrane by ImageJ. The cell number was shown by the average of triplicate assays for each experimental condition. Similar inserts coated with Matrigel were used to determine invasiveness of MDA-MB-231 cells in the invasion assay. A total of 3 × 10^4^ MDA-MB-231 cells (300 µl) after viral transduction in DMEM medium without FBS were added to the upper chamber. A total of 800 *μ*l complete medium was added to the bottom wells of the chambers. Then invasion cell numbers were compared at 24 h.

### Statistical analysis of experiment results

Data were presented as mean ± standard deviation of at least three individual experiments. Statistical analysis was performed by two tails Student’s *t* test or one-way ANOVA analysis by means of the Prism statistical software package (Graph Pad Software, Inc., La Jolla, CA, USA). *P < 0.05 was considered statistically significant.

### Reporting summary

Further information on research design is available in the Nature Research Reporting Summary linked to this article.

## Supplementary information

Supplementary Information

Description of Additional Supplementary Files

Supplementary Data 1

Supplementary Data 2

Supplementary Data 3

Supplementary Data 4

Supplementary Data 5

Supplementary Data 6

Supplementary Data 7

Supplementary Data 8

Supplementary Data 9

Supplementary Data 10

Supplementary Data 11

Supplementary Data 12

Reporting Summary

## Data Availability

The source of each dataset analyzed in this project was listed in the Supplementary Data 7. Source data are provided with this paper. Important processed data were deposited to https://figshare.com/projects/SuperRepressiveDomain/59723.

## Source data

Source Data
