# Supplementary material for: Broad genic repression domains signify enhanced silencing of oncogenes
Source: Nat Commun. 2020 Nov 3;11:5560. doi: 10.1038/s41467-020-18913-8 (PMC7641226; doi:10.1038/s41467-020-18913-8)
Supplement: Supplementary file 1 — Supplementary Information [file 41467_2020_18913_MOESM1_ESM.pdf]

SUPPLEMENTARY INFORMATION

Broad Genic Repression Domains Signify Enhanced Silencing of Oncogenes

Dongyu Zhao[1, 2, 3, 4, #], Lili Zhang[2, 3, 4, #, *], Min Zhang[2, 3, 4], Bo Xia[1, 2, 3, 4], Jie Lv[1, 2, 3, 4], Xinlei Gao[1, 2, 3, 4], Guangyu Wang[1, 2, 3, 4], Qingshu Meng[5, 6], Yang Yi[5, 6], Sen Zhu[1, 2, 3, 4], Alin S. Tomoiaga[7], Min Gyu Lee[8], John P. Cooke[2, 3, 4], Qi Cao[5, 6, *], Kaifu Chen[1, 2, 3, 4, *]

[1]Center for Bioinformatics and Computational Biology, [2]Center for Cardiovascular Regeneration, Department of Cardiovascular Sciences, Houston Methodist Research Institute, Houston, TX, USA. [3]Department of Cardiothoracic Surgeries, Weill Cornell Medical College, Cornell University, New York, NY, USA. [4]Institute for Academic Medicine, Houston Methodist Hospital, TX, USA. [5]Department of Urology, [6]Robert H. Lurie Comprehensive Cancer Center, Feinberg School of Medicine, Northwestern University, Chicago, IL, USA. [7]Business Analytics, CIS & Law Department, The O'Malley School of Business Accounting, Manhattan College, Riverdale, NY, USA. [8]Department of Molecular and Cellular Oncology, The University of Texas MD Anderson Cancer Center, Houston, TX, USA

# Equal contribution

*Corresponding to:
Kaifu Chen, kchen2@houstonmethodist.org
Qi Cao, qi.cao@northwestern.edu
Lili Zhang, lzhang3@houstonmethodist.org

Supplementary Figures 1-20

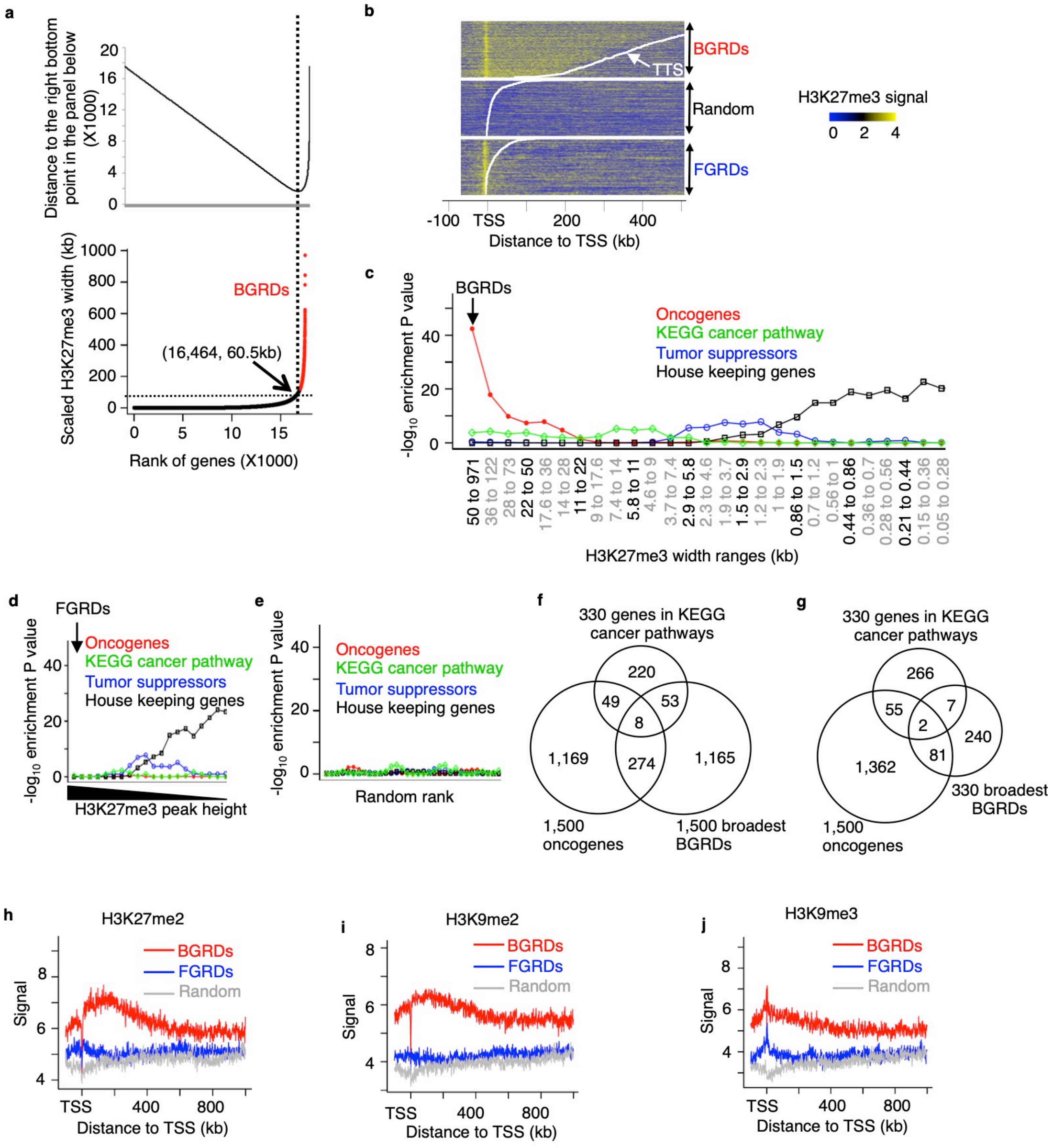

Supplementary Figure 1. Oncogenes reside in BGRDs on chromatin in normal CD4^{+} T cell.

(a) Cumulative plot of H3K27me3 width at individual genes (bottom panel). Genes were ranked by width of H3K27me3 from the narrowest at the left side of the x-axis to the broadest at the right side. The H3K27me3 width of each gene was then indicated on the y-axis (bottom). For each point in the curve of the bottom panel, distance to the right bottom point was indicated in the top panel. **(b)** Heatmaps to show H3K27me3 ChIP-Seq signal values at individual base pairs (columns) around TSS (rows) associated with BGRDs (top panel), random domains (middle panel), and FGRDs (bottom panel), respectively. White curve indicates TSS location of each gene. **(c-d)** Enrichment level of each gene category plotted against H3K27me3 peak width (c) or height (d). Genes were ranked by H3K27me3 width (c) or height (d) and divided into groups that each contains

1,500 genes, with two neighboring groups in the rank having 500 genes in common. A dot in each curve indicates the enrichment level (Y-axis) of one of these groups (X-axis) in the oncogenes, KEGG cancer pathway genes, tumor suppressor genes, or housekeeping genes as indicated by the color legends. The range of H3K27me3 width was indicated on the X-axis (c). **(e)** Enrichment level for each gene category plotted for individual randomly picked gene groups, of which each contains 1,500 genes. **(f, g)** Venn diagram to show the overlap among KEGG cancer pathway genes, TUSON oncogenes, and BGRD genes. **(h-j)** Average ChIP-Seq signal of H3K27me2 (h), H3K9me2 (i), and H3K9me3 (j) plotted around TSS associated with each category of repressive domains. P values determined by one tail Fisher's exact test (c, d, e).

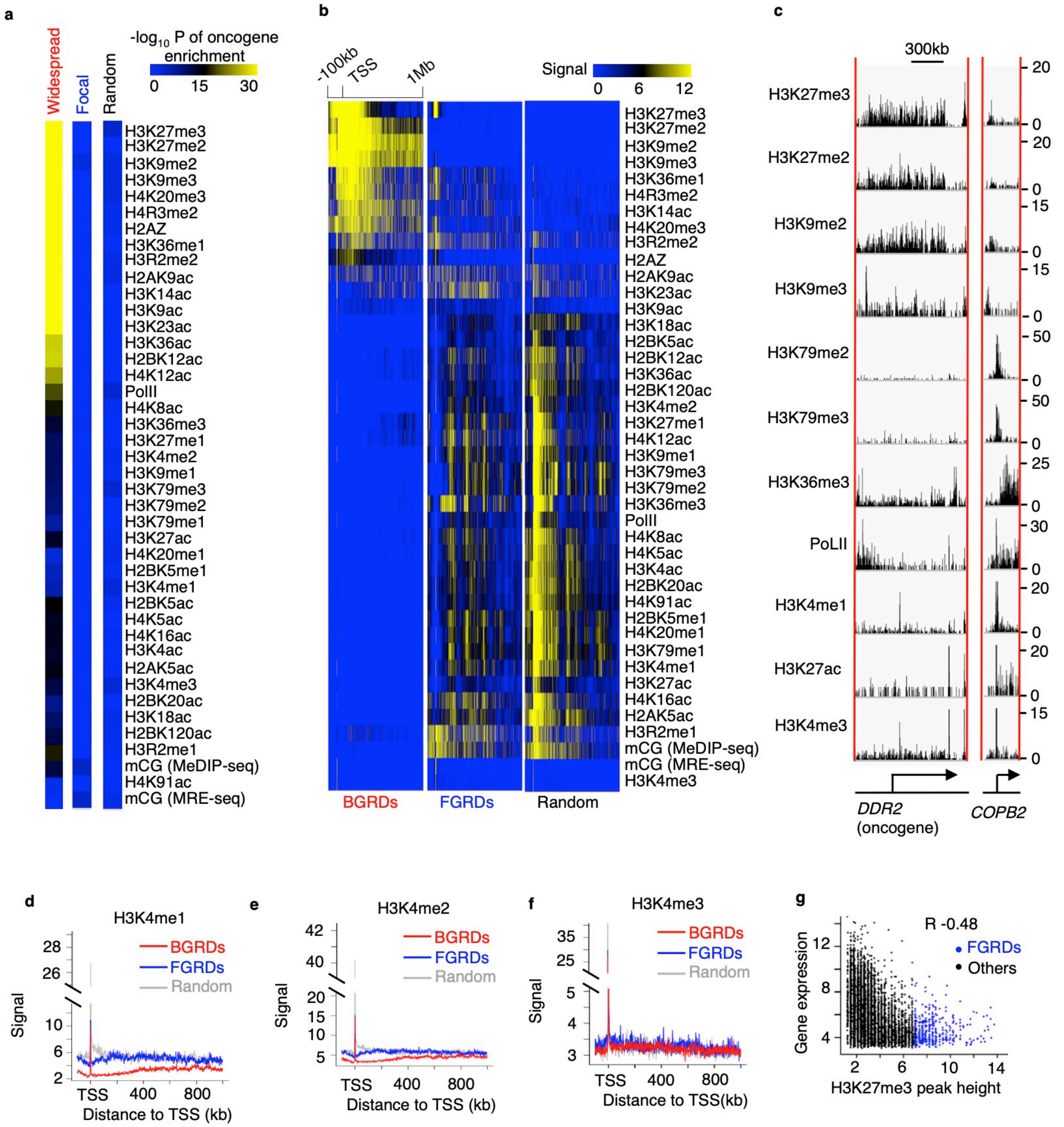

Supplementary Figure 2. Oncogenes also reside in BGRDs defined by other chromatin modifications in CD4^{+} T cell.

(a) Heatmap to show one tail Fisher's exact test P value for the significance of overlap between oncogenes and genes associated with widespread, focal, or random enrichment peaks of each chromatin mark, respectively. **(b)** Heatmap to show average signal value of each chromatin mark (row) at each base pair (column) around TSS of genes associated with BGRDs (left panel), FGRDs (middle panel), or random domains (right panel) defined by H3K27me3, respectively. **(c)** ChIP-Seq signal of individual chromatin marks at two example genes. Arrows indicate gene loci. Gene names were indicated at the bottom. Y-axis scale at the right side indicates ChIP-Seq signal strength. The scale of region length was indicated on the top. **(d-f)** Average ChIP-Seq signal value of H3K4me1 (d), H3K4me2 (e), and H3K4me3 (f) plotted around TSS associated with each category

of repressive domains. **(g)** Microarray expression value for each gene plotted against height of its associated H3K27me3 enrichment peak, with Spearman correlation coefficients indicated on top. Signal value for each epigenetic mark determined by ChIP-Seq except specifically indicated for mCG that were determined by MeDIP-Seq or MRE-Seq (a-c). Source data are provided as a Source Data file.

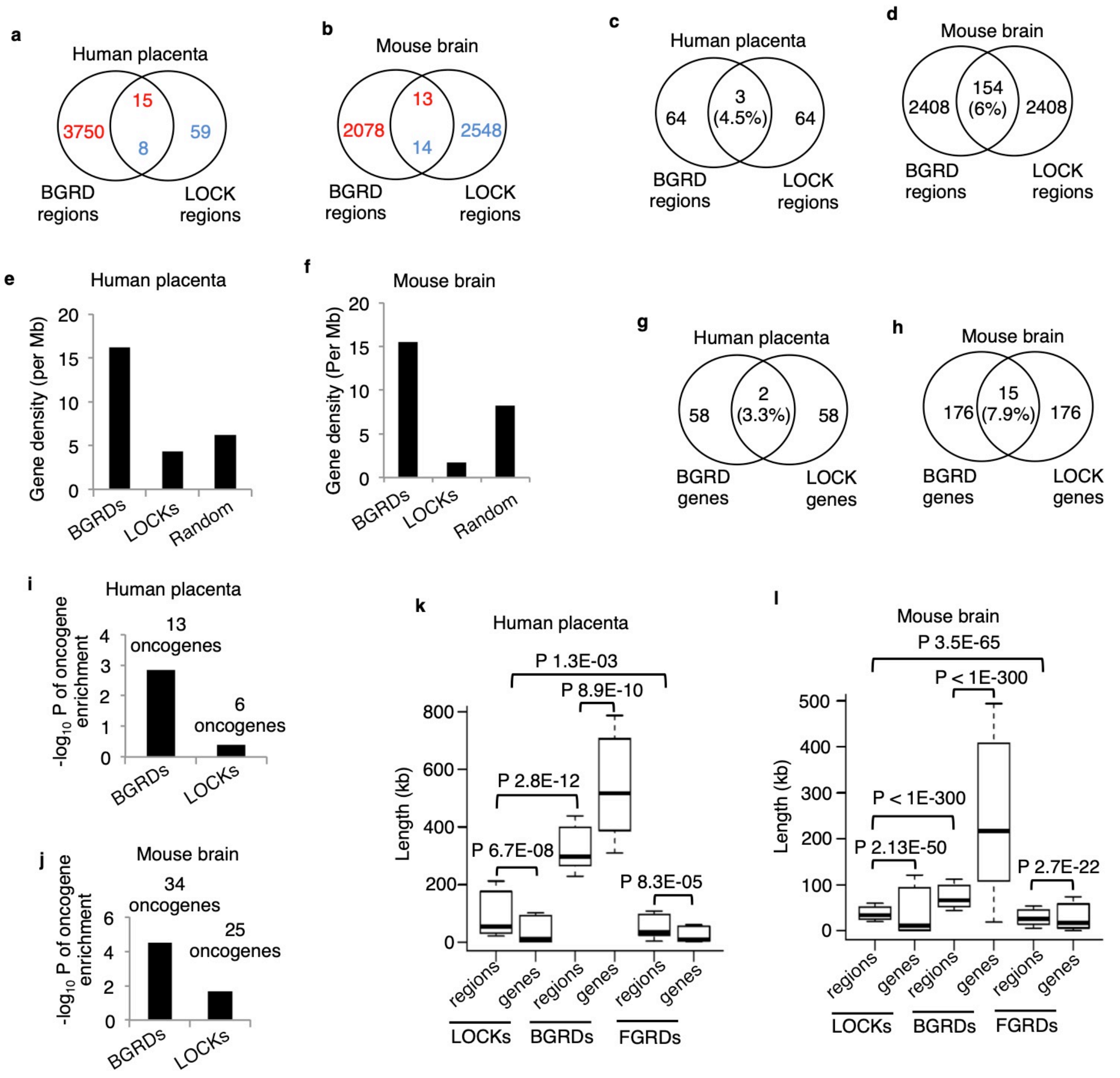

Supplementary Figure 3. BGRD, LOCK, and FGRD represent three different types of chromatin repression domains.

(a-d) Venn diagram to show the overlap between BGRDs and LOCKs in human placenta (a, c) and mouse brain (b, d) samples. Numbers of BRGDs and LOCKs were either the same (c, d) or not the same (a, b). **(e, f)** The density of genes in the same number of BGRDs and LOCKs. We ranked BGRDs and LOCKs by their sizes from large to small, respectively. Between each pair of BGRD and LOCK at the same rank, we redefined the shorter one by extending the two ends to the same length with the longer one. **(g, h)** Venn diagram to show the overlap between genes that reside in BGRDs and LOCKs in human placenta (g) and mouse brain (h) samples. Since there are only 60 (human placenta) and 191 (mouse brain) genes in LOCKs, the top 60 and 191 genes ranked by size of associated BGRDs were used for this analysis, respectively. **(i, j)** Enrichment level and the number of oncogenes in genes that reside in BGRDs and LOCKs. **(k, l)** Boxplot for length of LOCKs, BGRDs, FGRDs, and their associated genes in human placenta (k) and mouse brain (l) samples, n(human regions)=66, n(human genes)=60, n(mouse

regions)=2,340 and n(mouse genes)=191. Box plots: center line is median, boxes show first and third quartiles, whiskers extend to the most extreme data points that are no more than 1.5-fold of the interquartile range from the box. P values determined by one tail Wilcoxon test (k, l) and one tail Fisher's exact test (i, j).

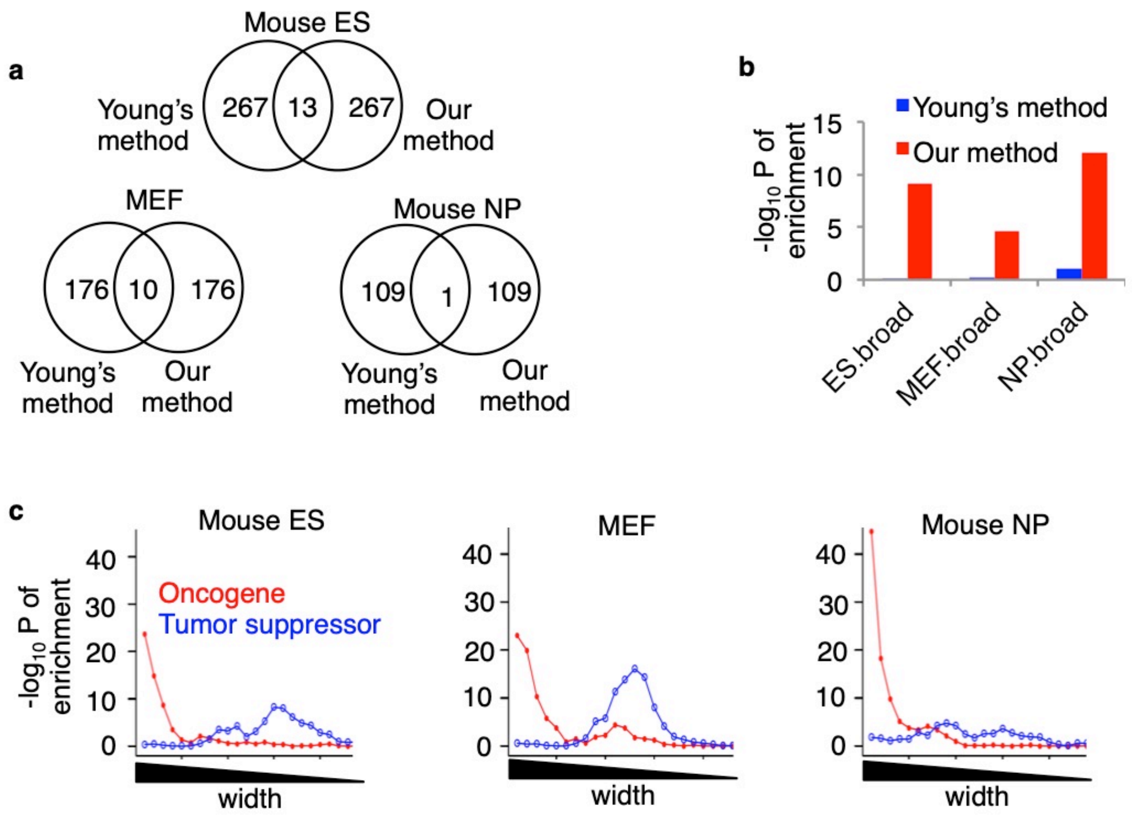

Supplementary Figure 4. Oncogenes were enriched in BGRDs, but not in Broad H3K27me3 defined by Young's method.

(a) Venn diagram to show the overlap between genes that display Broad H3K27me3 defined by Young's method in a recent literature and genes associated with BGRDs defined by our method. **(b)** Fisher's exact test P value of overlap between oncogenes and genes that display Broad H3K27me3 defined by Young's method in a recent literature or genes associated with BGRDs defined by our method. **(c)** Enrichment level and number of oncogenes or tumor suppressors plotted against H3K27me3 width determined by our method in three cell types from mouse. P values determined by one tail Fisher's exact test (b, c).

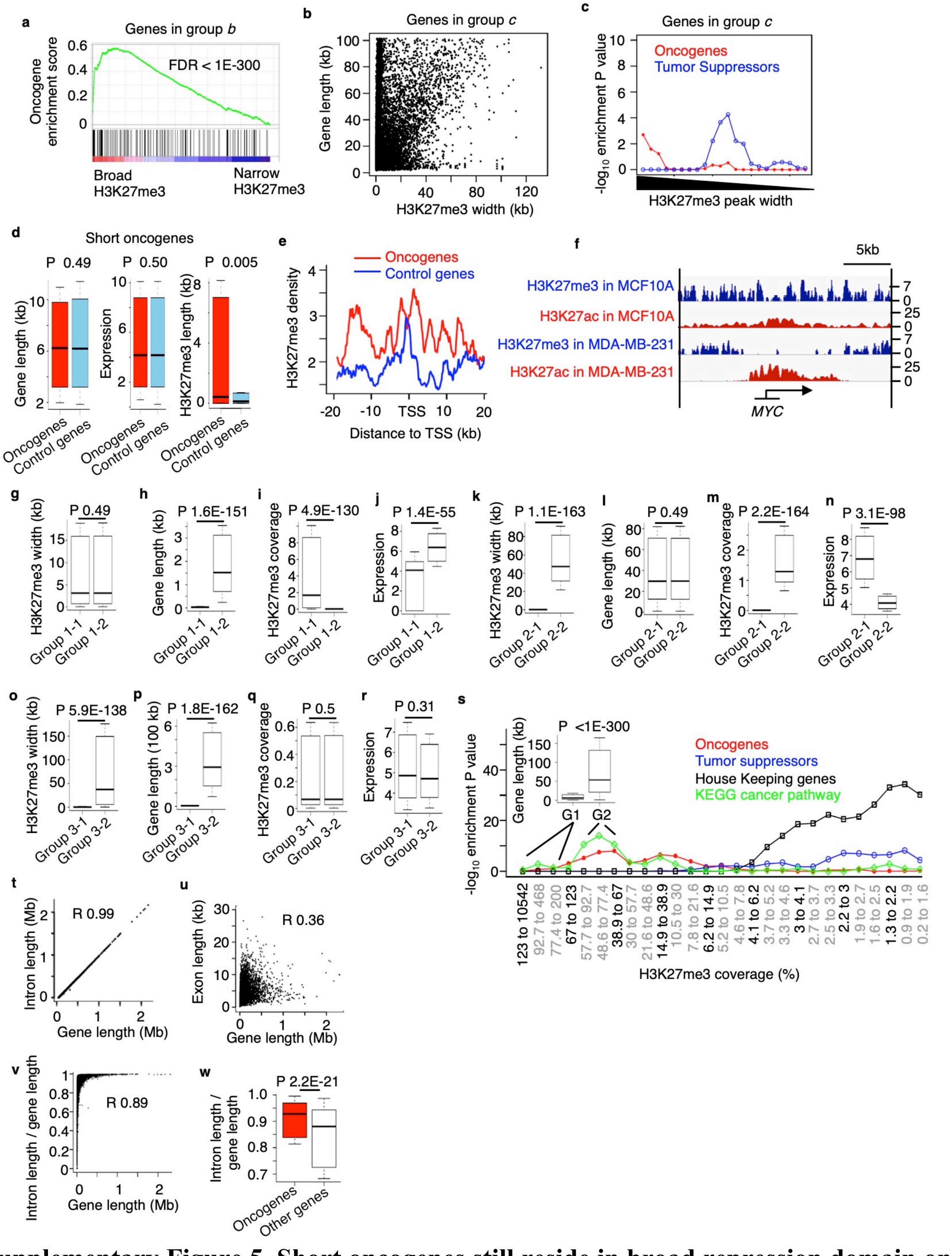

Supplementary Figure 5. Short oncogenes still reside in broad repression domain on chromatin in normal CD4^{+} T cell.

(a) GSEA analysis showing enrichment level of oncogenes in group *b* genes indicated in Fig. 2a. **(b)** Gene length plotted against width of associated repression domain for group *c* genes indicated in Fig. 2a. **(c)** Enrichment level of each gene category plotted against

H3K27me3 peak width of genes in the group *c* indicated in Fig. 2a. Genes were ranked by H3K27me3 width and divided into groups that each contains 1,500 genes, with two neighboring groups in the rank having 500 genes in common. A dot in each curve indicates the enrichment level (Y-axis) of one of these groups (X-axis) in the oncogenes or tumor suppressor genes as indicated by the color legends. **(d)** Boxplot to show lengths, expression values, and repression domain widths of the shortest 100 oncogenes and a set of 100 neutral control genes whose length and expression level are the closest to these short oncogenes. **(e)** H3K27me3 average ChIP-Seq signal around TSS of the shortest 100 oncogenes and the matched control genes as indicated in (d). **(f)** H3K27me3 and H3K27ac ChIP-Seq signal across the *MYC* locus. Arrows indicate gene loci associated with these domains. Y-axis scale at the right side indicates ChIP-Seq signal strength. **(g-r)** Boxplot for H3K27me3 width (g, k, o), gene length (h, l, p), H3K27me3 coverage (H3K27me3 width divided by gene length) (i, m, q), and gene expression (j, n, r) values for gene sets that were defined to have similar H3K27me3 width but different gene length (g-j), have the same gene length but different H3K27me3 width (k-n), have the same H3K27me3 coverage but different H3K27me3 width or gene length (o-r) in $CD4^+$ T cells. n=500 for each group. **(s)** Enrichment level of each gene category plotted against H3K27me3 coverage ranks. Genes were ranked by H3K27me3 coverage and divided into groups that each contains 1,500 genes, with two neighboring groups in the rank having 500 genes in common. A dot in each curve indicates the enrichment level (Y-axis) of one of these groups (X-axis) in the oncogenes, KEGG cancer pathway genes, tumor suppressor genes, or housekeeping genes as indicated by the color legends. The range of H3K27me3 coverage for each gene group was labeled on the X-axis. The boxplot shows gene length between groups with high and low H3K27me3 coverage. n=2500 for each group. **(t, u)** Intron length (t) and exon length (u) in each gene plotted against gene length. **(v)** Proportion of intron sequences in each gene plotted against gene length. **(w)** Boxplot to show proportion of intron sequences in oncogenes and other genes, n=500. Box plots: center line is median, boxes show first and third quartiles, whiskers extend to the most extreme data points that are no more than 1.5-fold of the interquartile range from the box (d, g-s, w). Spearman correlation coefficients were indicated (t-v). Gene expression values were calculated from microarray data. P values determined by one tail Wilcoxon test (d, g-s, w) and one tail Fisher's exact test (c, s). Source data are provided as a Source Data file.

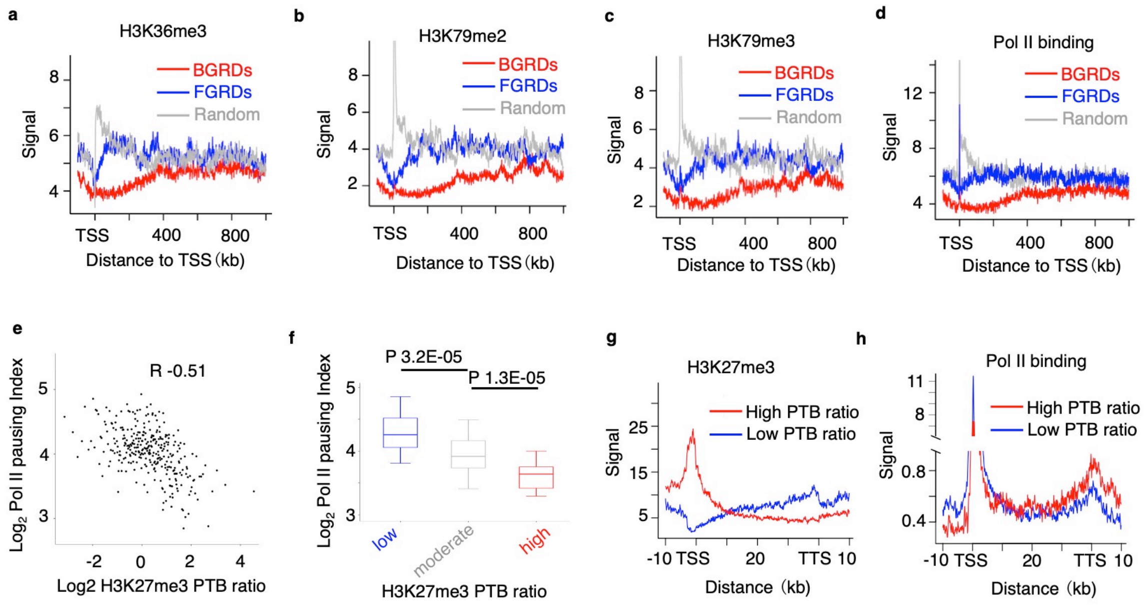

Supplementary Figure 6. Widespread H3K27me3 in CD4^{+} T cell BGRDs indicates constraint to both elongation and initiation stages of transcription.

(a-d) Average ChIP-Seq signal of H3K36me3 (a), H3K79me2 (b), H3K79me3 (c), and Polymerase II (d) plotted for each gene group around TSS. **(e)** Pol II pausing index plotted against H3K27me3 PTB ratio. Spearman correlation coefficient was indicated on top. Each dot represents an average of 20 genes that has the closest H3K27me3 PTB ratio. **(f)** Boxplot for Pol II pausing index values of genes associated with high, moderate, or low H3K27me3 PTB ratio, n=500 for each group. Box plots: center line is median, boxes show first and third quartiles, whiskers extend to the most extreme data points that are no more than 1.5-fold of the interquartile range from the box. P values calculated based on one tail Wilcoxon test. **(g, h)** Average ChIP-Seq signal of H3K27me3 (g) and Pol II (h) plotted for genes associated with high or low H3K27me3 PTB ratio. High, moderate, and low H3K27me3 PTB ratio (f) were defined to be that of the top 10% (~1200 genes), middle 10% and bottom 10% genes, respectively, after sorting genes by the H3K27me3 PTB ratio. P values determined by one tail Wilcoxon test. TSS: transcription start site; TTS: transcription terminate site.

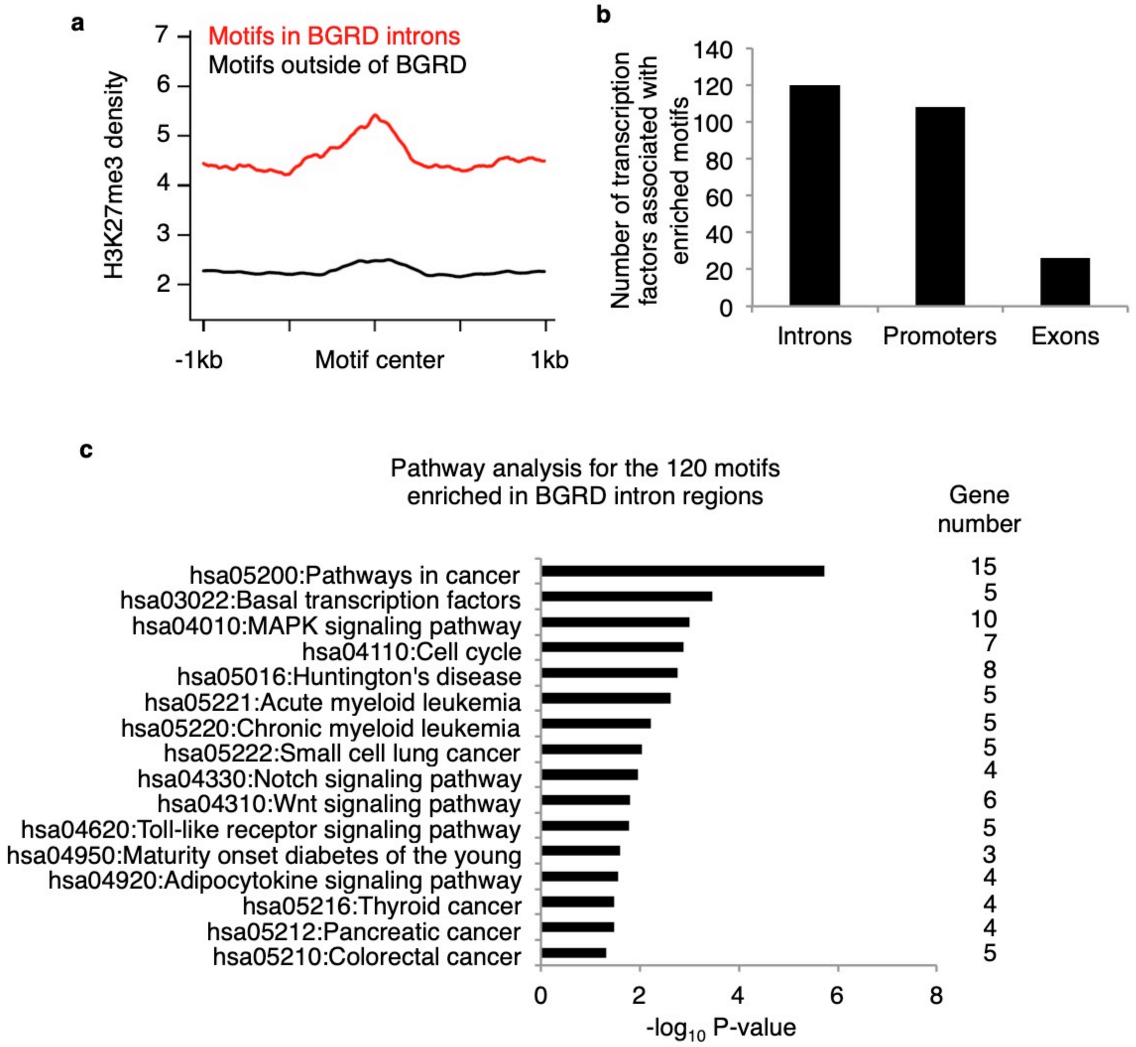

Supplementary Figure 7. Binding motifs for transcription factors in BGRD intron regions were enriched with H3K27me3 modification.

(a) Average ChIP-Seq signal value of H3K27me3 plotted around motif center. **(b)** Bar plot to show the number of transcription factors whose binding motifs were enriched in BGRD intron, promoter and exons regions, respectively. **(c)** Enrichment level of KEGG pathways in transcription factors whose binding motifs were enriched in BGRD intron regions. P values determined by one tail Fisher's exact test. Source data are provided as a Source Data file.

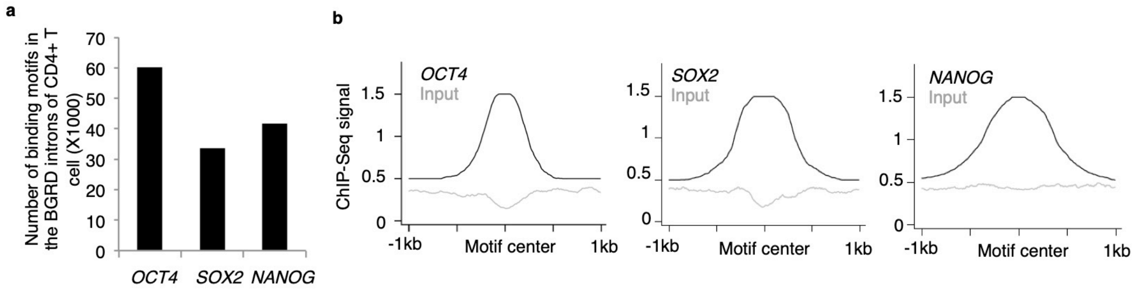

Supplementary Figure 8. Transcription factor binding motifs in BGRD introns of $CD4^+$ T cells could be bound by transcription factors in some other cell types.

(a) Bar plot to show the number of binding motifs for transcription factors *OCT4*, *SOX2* and *NANOG* in BGRD intron regions of $CD4^+$ T cell. **(b)** Average ChIP-Seq signal value for *OCT4*, *SOX2* and *NANOG* in stem cells plotted around center of their binding motifs in BGRD intron regions of $CD4^+$ T cells.

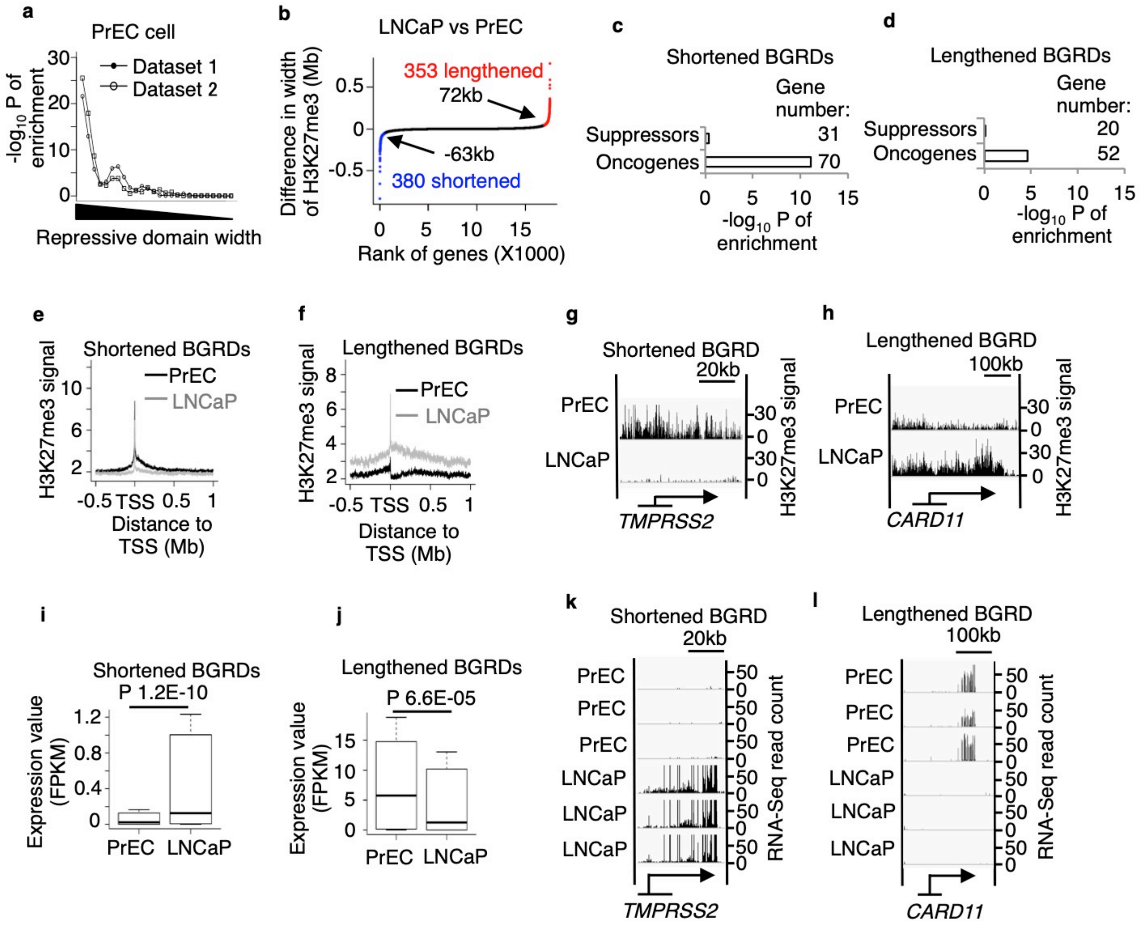

Supplementary Figure 9. Shortening of BGRDs is associated with up regulation of oncogenes in prostate cancer cells.

(a) Enrichment level of oncogenes plotted against width of repressive domain in prostate epithelial cells. **(b)** Cumulative plot of difference in H3K27me3 width between the cells LNCaP and PrEC. Genes were ranked by difference in H3K27me3 width from the most shortened at the left side of x-axis to the most lengthened at the right side. **(c, d)** Enrichment level of oncogenes or tumor suppressors in genes that show shortening (c) or lengthening (d) of BGRDs in LNCaP relative to PrEC cells. **(e, f)** Average ChIP-Seq signal of H3K27me3 plotted around TSS of genes with shortened (e) or lengthened (f) BGRDs in LNCaP relative to PrEC cells. **(g, h)** ChIP-Seq signal of H3K27me3 at example genes in PrEC and LNCaP cells. Arrows indicate gene loci. Gene names were indicated at the bottom. Y-axis scale at the right side indicates ChIP-Seq signal strength. The scale of region size was indicated on the top. **(i, j)** Boxplot for expression values of genes with shortened (i) or lengthened (j) BGRDs in LNCaP relative to PrEC cells, n(lengthened)=353 and n(shortened)=380. Box plots: center line is median, boxes show first and third quartiles, whiskers extend to the most extreme data points that are no more than 1.5-fold of the interquartile range from the box. **(k, l)** RNA-Seq read counts at example genes that display shortening (k) or lengthening (l) of BGRDs in LNCaP relative to PrEC cells. Arrows indicate gene loci. Gene names were indicated at the bottom. Y-

axis scale at the right side indicates RNA-Seq signal strength. The scale of region length was indicated on the top. P values determined by one tail Fisher's exact test (a, c, d) or one tail Wilcoxon test (i, j).

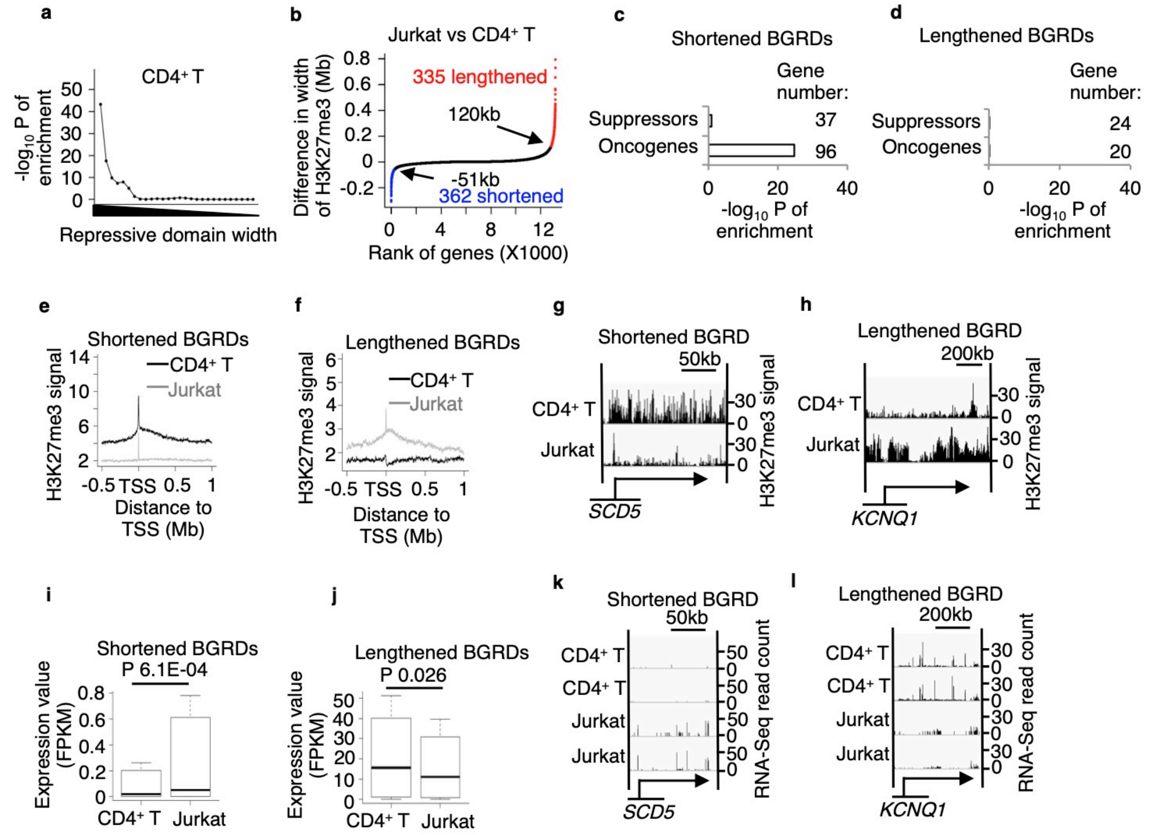

Supplementary Figure 10. Shortening of BGRDs is associated with up regulation of oncogenes in leukemia cells.

(a) Enrichment level of oncogenes plotted against width of repressive domain in $CD4^+$ T cell. **(b)** Cumulative plot of difference in H3K27me3 width between the Jurkat cells and $CD4^+$ T cell. Genes were ranked by difference in H3K27me3 width from the most shortened at the left side of x-axis to the most lengthened at the right side. **(c, d)** Enrichment level of oncogenes or tumor suppressors in genes that show shortening (c) or lengthening (d) of BGRDs in Jurkat cells relative to $CD4^+$ T cells. **(e, f)** Average ChIP-Seq signal of H3K27me3 plotted around TSS of genes with shortened (e) or lengthened (f) BGRDs in Jurkat cells relative to $CD4^+$ T cells. **(g, h)** ChIP-Seq signal of H3K27me3 at example genes in Jurkat and $CD4^+$ T cells. Arrows indicate gene loci. Gene names were indicated at the bottom. Y-axis scale at the right side indicates ChIP-Seq signal strength. The scale of region length was indicated on the top. **(i, j)** Boxplot for expression values of genes with shortened (i) or lengthened (j) BGRDs in Jurkat cells relative to $CD4^+$ T cells, n(lengthened)=335 and n(shortened)=362. Box plots: center line is median, boxes show first and third quartiles, whiskers extend to the most extreme data points that are no more than 1.5-fold of the interquartile range from the box. (**k, l**) RNA-Seq read count at example genes that display shortening (k) or lengthening (l) of BGRDs in Jurkat cells relative to $CD4^+$ T cells. Arrows indicate gene loci. Gene names were indicated at the bottom. Y-axis scale at the right side indicates RNA-Seq signal strength. The scale of

region length was indicated on the top. P values determined by one tail Fisher's exact test (a, c, d) or one tail Wilcoxon test (i, j).

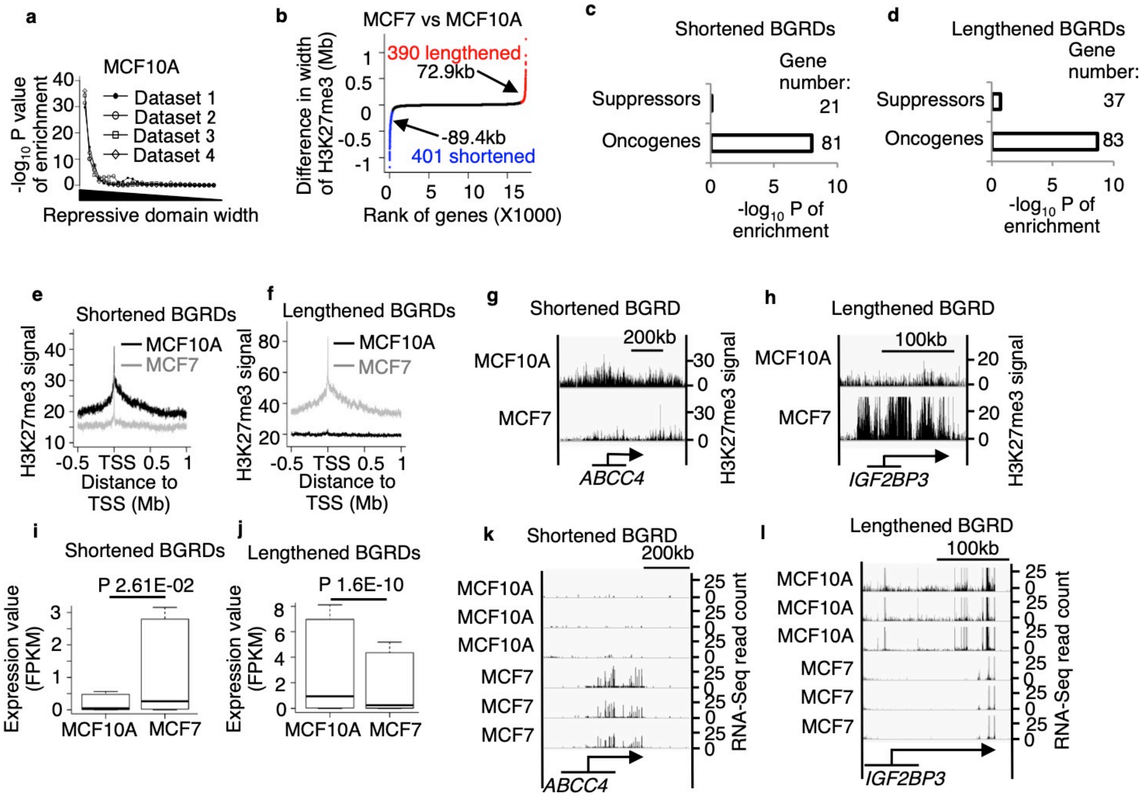

Supplementary Figure 11. Disruption of BGRDs in breast cancer cell line MCF7 relative to normal breast epithelial cell line MCF10A is associated with up regulation of oncogenes.

(a) Enrichment level of oncogenes plotted against width of repression domain in breast epithelial cell lines (The same as Fig. 5a, showing here again for consistency of figure panels). Genes were ranked by H3K27me3 width and divided into groups that each contains 1,500 genes, with two neighboring groups in the rank having 500 genes in common. A dot in each curve indicates the enrichment level (Y-axis) of one of these groups (X-axis) in the oncogenes. **(b)** Cumulative plot of difference in H3K27me3 width between the MCF7 cells and MCF10A cell. Genes were ranked by difference in H3K27me3 width from the most shortened at the left side of x-axis to the most lengthened at the right side. **(c, d)** Enrichment level of oncogenes or tumor suppressors in genes that show shortening (c) or lengthening (d) of BGRDs. **(e, f)** Average H3K27me3 signal around TSS of genes that show shortening (e) or lengthening (f) of BGRDs. **(g, h)** ChIP-Seq signal of H3K27me3 at example genes. **(i, j)** Boxplot for expression values of genes that show shortening (i) or lengthening (j) of BGRDs, n(lengthened)=390 and n(shortened)=401. Box plots: center line is median, boxes show first and third quartiles, whiskers extend to the most extreme data points that are no more than 1.5-fold of the interquartile range from the box. **(k, l)** RNA-Seq read count at example genes that display shortening (k) or lengthening (l) of BGRDs. P values determined by one tail Fisher's exact test (a, c, d) or one tail Wilcoxon test (i, j).

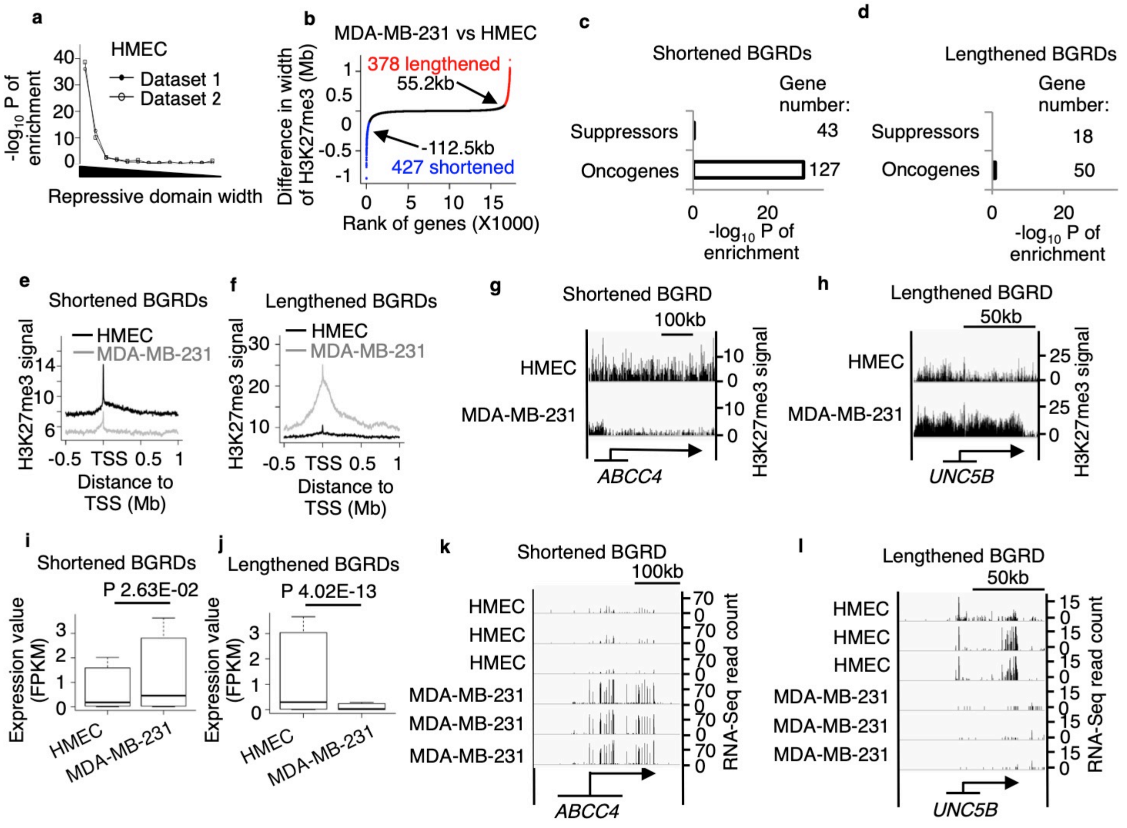

Supplementary Figure 12. Disruption of BGRDs in breast cancer cell line MDA-MB-231 relative to primary mammary epithelial cells HMEC is associated with up regulation of oncogenes.

(a) Enrichment level of oncogenes plotted against width of repressive domain in primary mammary epithelial cells (HMEC). **(b)** Cumulative plot of difference in H3K27me3 width between the cells MDA-MB-231 and HMEC. Genes were ranked by difference in H3K27me3 width from the most shortened at the left side of x-axis to the most lengthened at the right side. **(c, d)** Enrichment level of oncogenes or tumor suppressors in genes that show shortening (c) or lengthening (d) of BGRDs. **(e, f)** Average H3K27me3 signal around TSS of genes that show shortening (e) or lengthening (f) of BGRDs. **(g, h)** ChIP-Seq signal of H3K27me3 at example genes. **(i, j)** Boxplot for expression values of genes that show shortening (i) or lengthening (j) of BGRDs, n(lengthened)=378 and n(shortened)=427. Box plots: center line is median, boxes show first and third quartiles, whiskers extend to the most extreme data points that are no more than 1.5-fold of the interquartile range from the box. **(k, l)** RNA-Seq read count at example genes that display shortening (k) or lengthening (l) of BGRDs. P values were determined by one tail Fisher's exact test (a, c, d) or one tail Wilcoxon test (i, j).

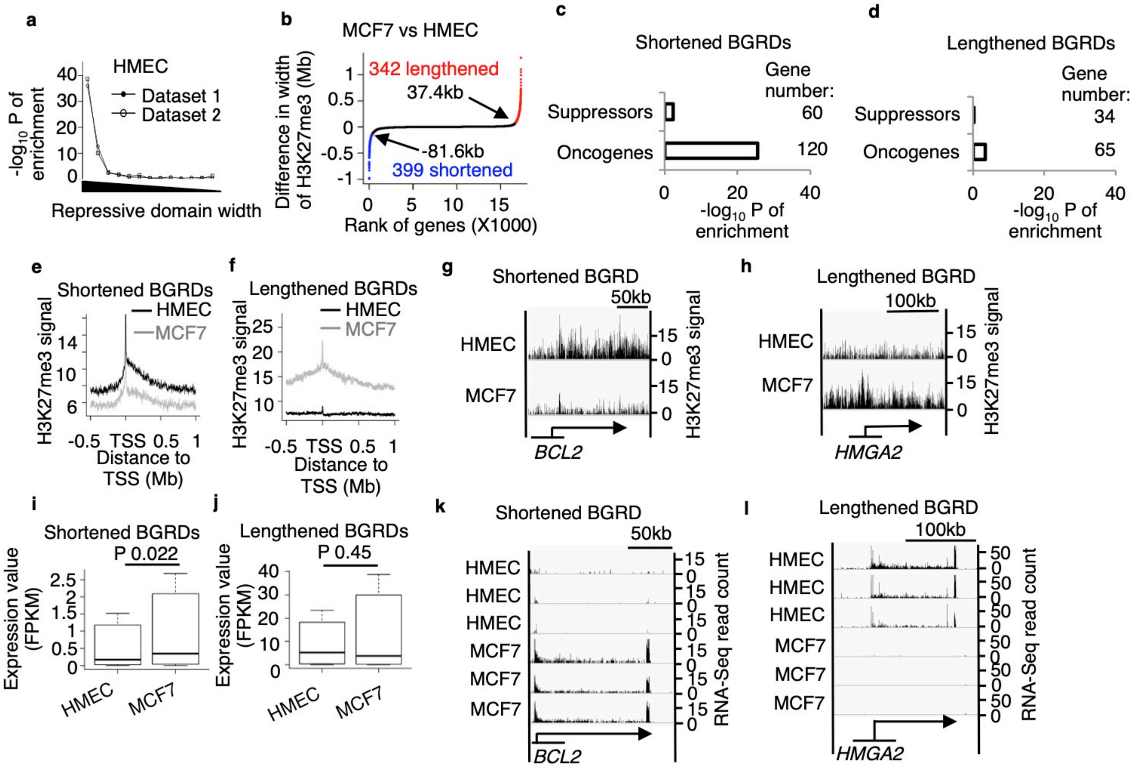

Supplementary Figure 13. Disruption of BGRDs in breast cancer cell line MCF7 relative to primary mammary epithelial cells HMEC is associated with up regulation of oncogenes.

(a) Enrichment level of oncogenes plotted against width of repressive domain in HMEC (The same as Supplementary Fig. 12a, showing here again for consistency of figure panels). **(b)** Cumulative plot of difference in H3K27me3 width between the cells MCF7 and HMEC. Genes were ranked by difference in H3K27me3 width from the most shortened at the left side of x-axis to the most lengthened at the right side. **(c, d)** Enrichment level of oncogenes or tumor suppressors in genes that show shortening (c) or lengthening (d) of BGRDs. **(e, f)** Average H3K27me3 signal around TSS of genes that show shortening (e) or lengthening (f) of BGRDs. **(g, h)** ChIP-Seq signal of H3K27me3 at example genes. **(i, j)** Boxplot for expression values of genes that show shortening (i) or lengthening (j) of BGRDs, n(lengthened)=342 and n(shortened)=399. Box plots: center line is median, boxes show first and third quartiles, whiskers extend to the most extreme data points that are no more than 1.5-fold of the interquartile range from the box. **(k, l)** RNA-Seq read count at example genes that display shortening (k) or lengthening (l) of BGRDs. P values were determined by one tail Fisher's exact test (a, c, d) or one tail Wilcoxon test (i, j).

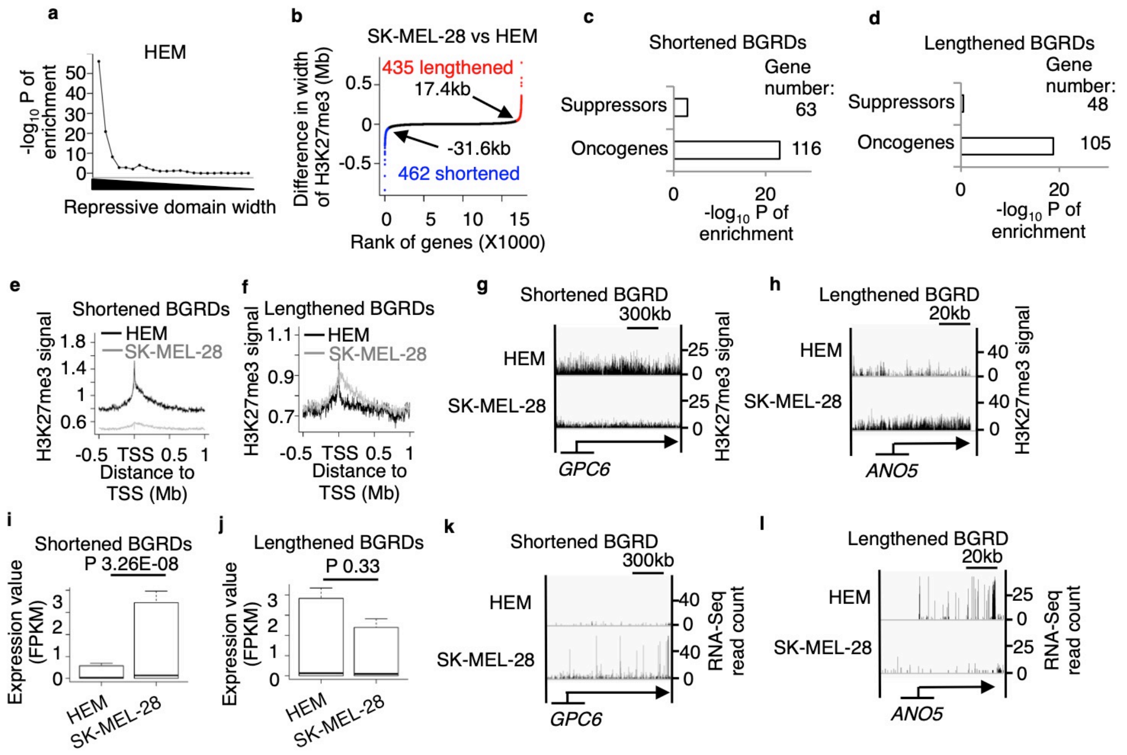

Supplementary Figure 14. Disruption of SRDs is associated with up regulation of oncogenes in melanoma cell relative to Human Epidermal Melanocytes.
(a) Enrichment level of oncogenes plotted against width of repressive domain in HEM. **(b)** Cumulative plot of difference in H3K27me3 width between the melanoma cell line SK-MEL-28 and Human Epidermal Melanocytes (HEM). Genes were ranked by difference in H3K27me3 width from the most shortened at the left side of x-axis to the most lengthened at the right side. **(c, d)** Enrichment level of oncogenes or tumor suppressors in genes that show shortening (c) or lengthening (d) of SRDs. **(e, f)** Average H3K27me3 signal around TSSs of genes that show shortening (e) or lengthening (f) of SRDs. **(g, h)** ChIP-Seq signal of H3K27me3 at example genes in the SK-MEL-28 and HEM. **(i, j)** Boxplot for expression values of genes that show shortening (i) or lengthening (j) of SRDs, n(lengthened)=435 and n(shortened)=462. Box plots: center line is median, boxes show first and third quartiles, whiskers extend to the most extreme data points that are no more than 1.5-fold of the interquartile range from the box. **(k, l)** RNA-Seq read count at example genes that display shortening (k) or lengthening (l) of SRDs. P values determined by one tail Fisher's exact test (a, c, d) or one tail Wilcoxon test (i, j).

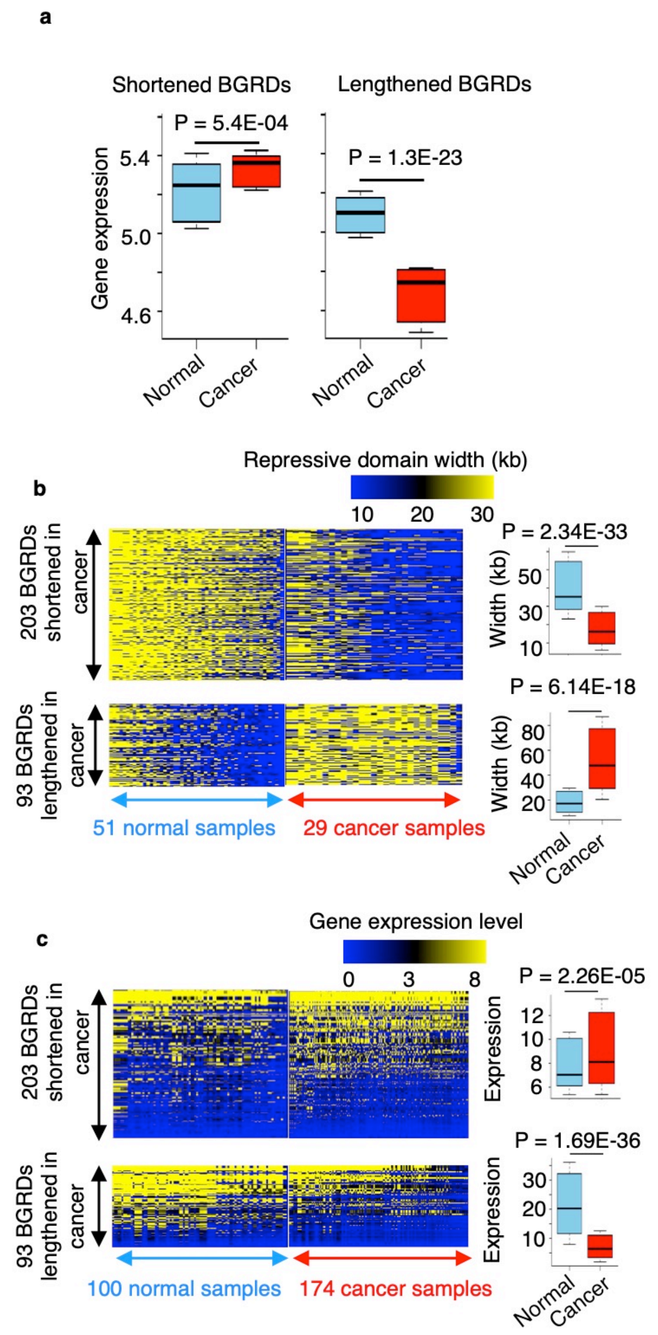

Supplementary Figure 15. Shortening of BGRDs is significantly associated with up regulation of oncogenes in pan-cancer analysis.
(a) Boxplot to show expression values determined by microarray data for gene groups associated with shortened or lengthened BGRDs in ENCODE cancer cells relative to normal cells, n(shortened)=203 and n(lengthened)=93. **(b)** Heatmap to show widths of repressive domains at individual genes (row) in individual samples (columns), with boxplots at the right side to further indicate difference in widths of repressive domains

between normal and cancer samples. Genes that display lengthening or shortening of BGRDs in 84 cancer cell samples relative to 113 non-cancer cell samples were presented. Only cell samples that have both ChIP-Seq data for H3K27me3 and RNA-Seq data were presented. **(c)** Heatmap to show expression values (FPKM) of individual genes (row) in individual samples (columns), with boxplots at the right side to further indicate difference in gene expression values between normal and cancer samples. Genes that display lengthening or shortening of BGRDs in 84 cancer cell samples relative to 113 non-cancer cell samples were presented. Only cell samples that have both ChIP-Seq data for H3K27me3 and RNA-Seq data were presented. Box plots: center line is median, boxes show first and third quartiles, whiskers extend to the most extreme data points that are no more than 1.5-fold of the interquartile range from the box (a-c). P values were determined by one tail Wilcoxon test. Source data are provided as a Source Data file.

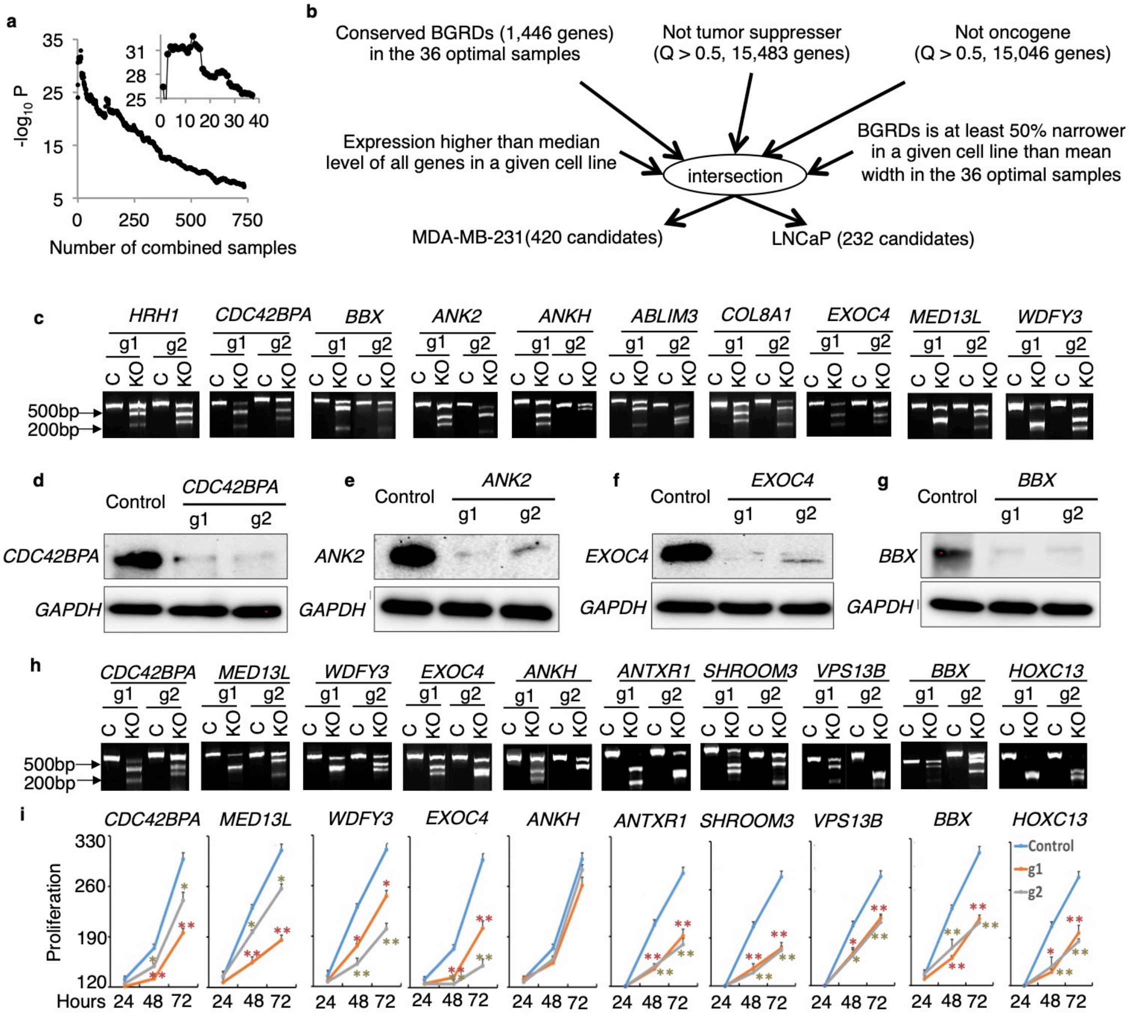

Supplementary Figure 16. A computational epigenetics pipeline to define putative tumor-promoting genes that are epigenetically altered in individual cancers.

(a) Enrichment level of oncogenes in genes associated with BGRDs plotted against number of optimal samples that were used to define the BGRDs. Genes associated with BGRDs in each used sample were combined to form a non-redundant gene list, for which the P value of the overlap with oncogenes was then determined by one tail Fisher's exact test. **(b)** The flowchart to define putative tumor-promoting genes by BGRD shortening in a cancer cell relative to an optimal combination of 36 samples. **(c)** T7 Endonuclease cleavage assay was performed to confirm cutting efficiency of each guide RNA for breast cancer cell MDA-MB-231 with each putative oncogene disrupted by CRISPR-Cas9 guide RNA g1, g2, or under control condition. **(d-g)** Western Blot results to verify CRISPR editing efficiency at individual genes in MDA-MB-231 cells. **(h)** T7 Endonuclease cleavage assay to confirm cutting efficiency of each guide RNA in LNCaP cell. (**i**) Proliferation rates plotted at 3 time points for prostate cancer cell LNCaP under control condition or with each putative oncogene disrupted by CRISPR-Cas9 guide RNA g1 or g2. All the data were analyzed from at least three independent experiments and the

quantification data were presented as mean ± SD. P values determined by one tail Fisher's exact test (a) or two tails Student's t test (i) and the exact P values were listed in the Source Data file; ***, $P < 0.001$; **, $P < 0.01$; *, $P<0.05$; n=5 independent experiments. Source data are provided as a Source Data file.

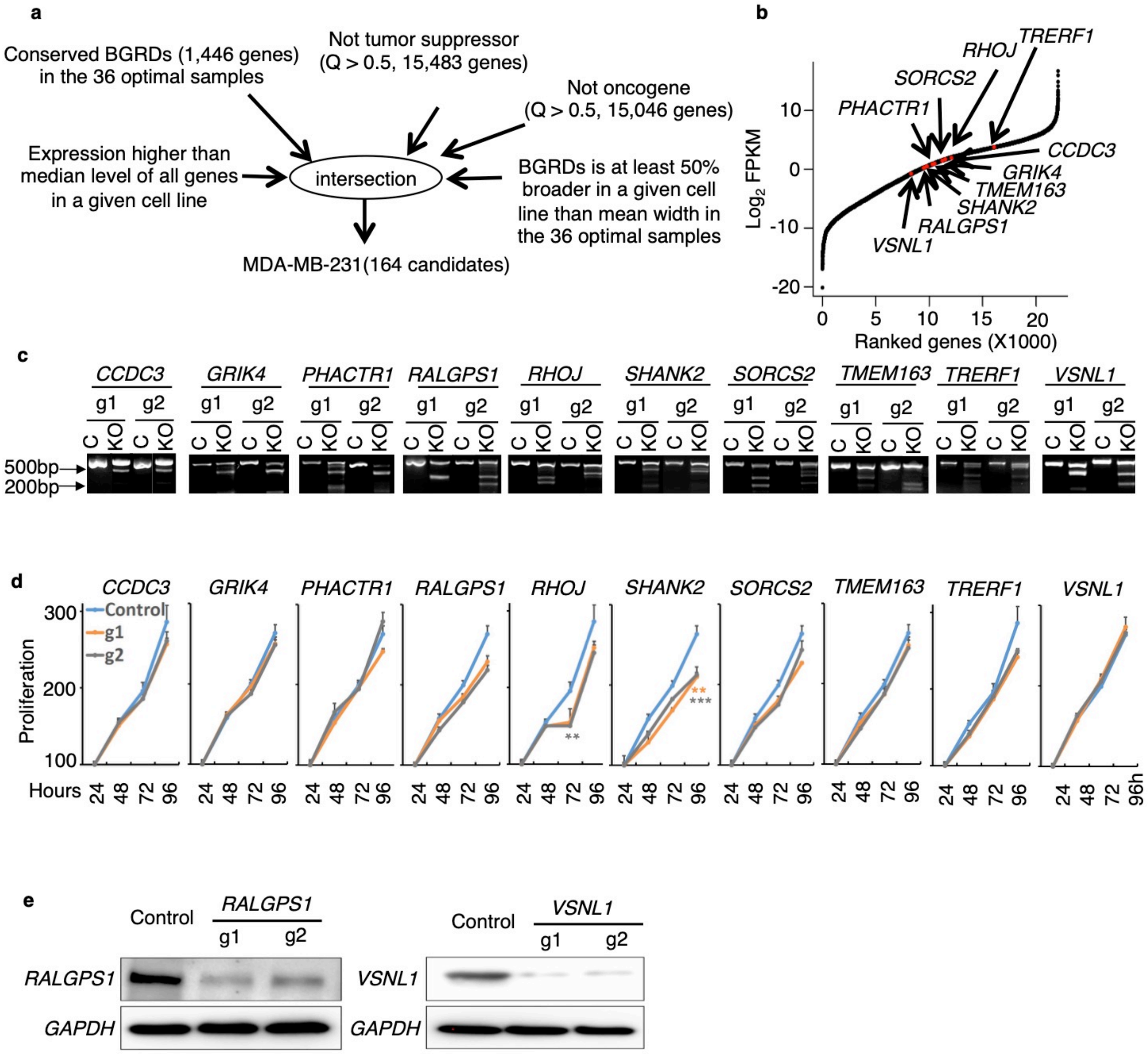

Supplementary Figure 17. A computational epigenetics pipeline to define genes associated with BGRD lengthening in breast cancer.

(a) The flowchart to define genes associated with BGRD lengthening in breast cancer cell MDA-MB-231. **(b)** Commutative plot to show expression value of individual genes in MDA-MB-231 cell. **(c)** T7 Endonuclease cleavage assay to confirm cutting efficiency of each guide RNA in MDA-MB-231 cell. **(d)** Proliferation rate of breast cancer cell MDA-MB-231 with individual genes disrupted by CRISPR-Cas9 guide RNA g1, g2, or under control condition. n=5 biologically independent experiments. **(e)** The protein level of *RALGPS1* and *VSNL1* in control and 2 CRISPR knockdown groups in MDA-MB-231 cell. All the data were analyzed from at least three independent experiments and the quantification data were presented as mean ± SD. P values determined by two tails Student's t test (d) and the exact P values were listed in the Source Data file; ***, P < 0.001; **, P < 0.01; *, P<0.05. Source data are provided as a Source Data file.

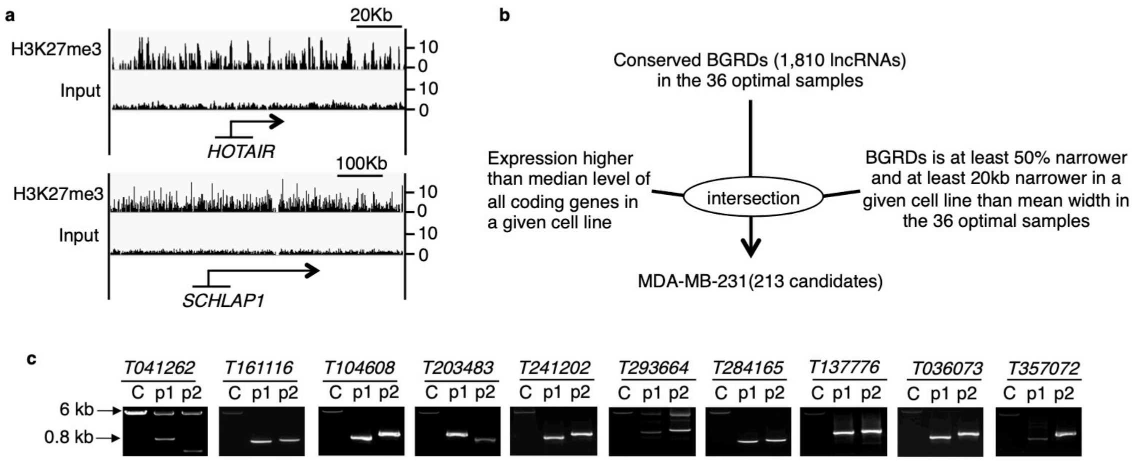

Supplementary Figure 18. A computational epigenetics pipeline to define putative tumor-promoting lncRNAs that were epigenetically altered in breast cancer.
(a) H3K27me3 ChIP-Seq signal and input signal in MCF10A cell across two well-known onco-lncRNAs. Arrows indicate lncRNA loci; lncRNA names were indicated at the bottom. Y-axis scale at the right side indicates ChIP-Seq signal strength. **(b)** The flowchart to define putative tumor-promoting lncRNAs for breast cancer cell MDA-MB-231 on the basis of BGRD shortening. **(c)** Knockout efficiency at DNA level was determined by PCR in breast cancer cell MDA-MB-231 with each candidate tumor-promoting lncRNA disrupted by CRISPR-Cas9 guide RNA pairs p1, p2, or under control condition. The experiments were repeated three times independently with similar results. Source data are provided as a Source Data file.

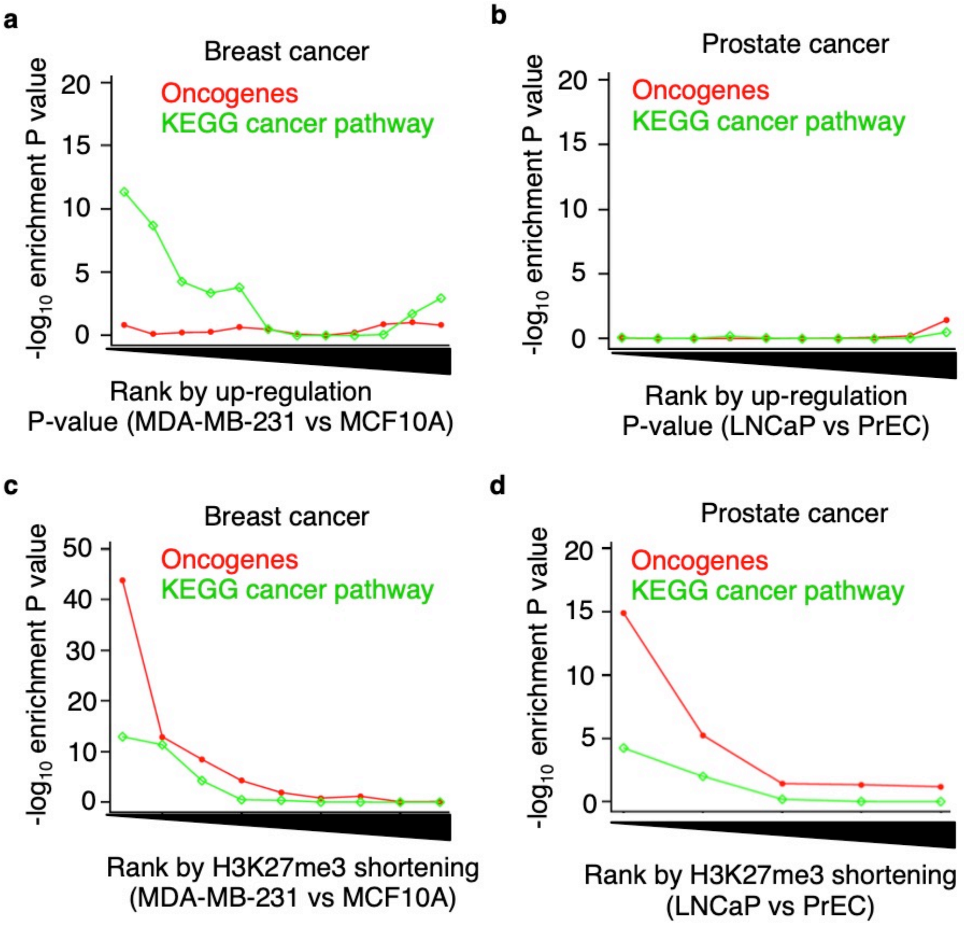

Supplementary Figure 19. Oncogenes show stronger enrichment at genes displaying change of BGRD length than at genes displaying change of expression in cancer cells relative to matched non-cancer cells.

(a, b) Enrichment level of genes in each gene category plotted against rank of gene expression (RNA-seq) up-regulation in breast (a) and prostate (b) cancer cells relative to non-cancer cells. Genes were ranked by p value of expression up-regulation and divided into groups that each contain 1,500 genes, with two neighboring groups in the rank having 500 genes in common. A dot in each curve indicates the enrichment level (Y-axis) of one of these groups (X-axis) in the oncogenes and KEGG cancer pathway genes, as indicated by the color legends. **(c, d)** Enrichment level of genes in each gene category plotted against rank of H3K27me3 width shortening in breast (c) and prostate (d) cancer cells relative to non-cancer cell. Genes were ranked by length of H3K27me3 shortening and divided into groups that each contains 1,500 genes, with two neighboring groups in the rank having 500 genes in common. A dot in each curve indicates the enrichment level (Y-axis) of one of these groups (X-axis) in the oncogenes and KEGG cancer pathway genes, as indicated by the color legends. P values determined by one tail Fisher's exact test.

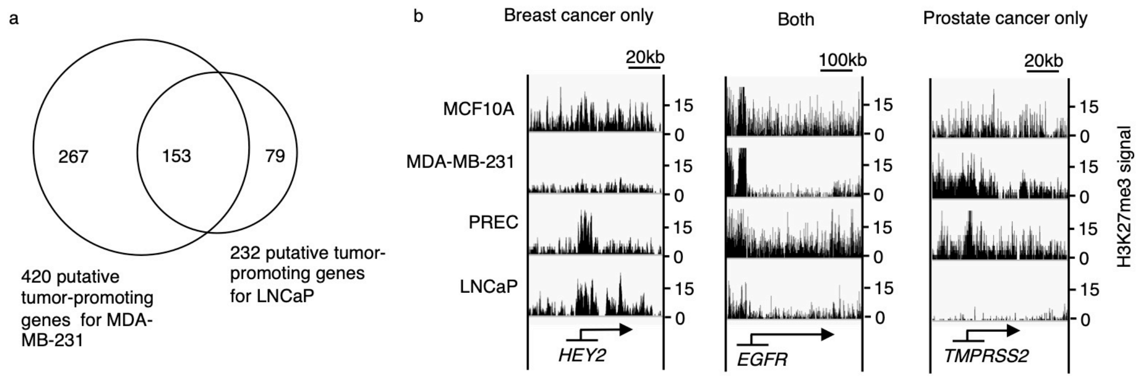

Supplementary Figure 20. Different cancer cells may show BGRD shortening at either a common subset or a cancer type-specific subset of genes.
(a) Venn diagram to show the overlap between 420 putative tumor-promoting genes for breast cancer and 232 putative tumor-promoting genes for prostate cancer that are identified based on our pipeline. **(b)** H3K27me3 ChIP-Seq signal at 3 reported oncogenes. Arrows indicate gene loci associated with these repressive domains. Gene names were indicated at the bottom side. Y-axis scale at the right side indicates ChIP-Seq signal strength.

Supplementary Table 1. 36 optimal datasets are used for recapturing tumor-promoting genes.

Database	Sample ID	Biosample	Cancer
ENCODE	ENCFF000BIR	Mammary epithelial cell	No
ENCODE	ENCFF002AUY	Neural cell	No
ENCODE	ENCFF023EPH	MCF-7	Yes
ENCODE	ENCFF094MAV	Loucy	Yes
ENCODE	ENCFF134NCG	MCF-7	Yes
ENCODE	ENCFF165RVV	Mid-neurogenesis radial glial cells	No
ENCODE	ENCFF172DNR	CD4-positive helper T cell	No
ENCODE	ENCFF217VZC	Thyroid gland	No
ENCODE	ENCFF250WAH	MCF-7	Yes
ENCODE	ENCFF381CEG	Thoracic aorta	No
ENCODE	ENCFF388UZO	Thyroid gland	No
ENCODE	ENCFF504LCB	Body of pancreas	No
ENCODE	ENCFF556ZYP	Thyroid gland	No
ENCODE	ENCFF590CFV	Loucy	Yes
ENCODE	ENCFF738ERP	MCF-7	Yes
ENCODE	ENCFF830PWL	Ascending aorta	No
GEO	GSM1023610	HGFDFN168	No
GEO	GSM1023612	HGFDFN168	No
GEO	GSM1217937	Pancreatic islets	No
GEO	GSM1217938	Pancreatic islets	No
GEO	GSM1217940	Pancreatic islets	No
GEO	GSM1217941	Pancreatic islets	No
GEO	GSM1217943	Pancreatic islets	No
GEO	GSM1217947	Pancreatic islets	No
GEO	GSM1217948	Pancreatic islets	No
GEO	GSM1217953	Pancreatic islets	No
GEO	GSM1372861	Endometrial stromal cells	No
GEO	GSM1436880	ESCs	No
GEO	GSM1697669	iPSCs	No
GEO	GSM1697672	iPSCs	No
GEO	GSM1697675	iPSCs	No
GEO	GSM1958046	MutuI	No
GEO	GSM2305249	LNCaP cells	Yes
GEO	GSM2305250	LNCaP cells	Yes
GEO	GSM641823	Epithelial cell	No
GEO	GSM641825	Epithelial cell	No
