# Supplementary material for: Broad genic repression domains signify enhanced silencing of oncogenes
Source: Nat Commun. 2020 Nov 3;11:5560. doi: 10.1038/s41467-020-18913-8 (PMC7641226; doi:10.1038/s41467-020-18913-8)
Supplement: Supplementary file 2 — Description of Additional Supplementary Files [file 41467_2020_18913_MOESM2_ESM.pdf]

Description of Additional Supplementary Files

Title: Supplementary Data 1.

Description: Enrichment of oncogenes, tumor suppressors, housekeeping genes, or KEGG cancer pathways genes in individual gene groups defined by rank of H3K27me3 peak width (a), height (b), or random ranks (c) in CD4+ T cell.

Title: Supplementary Data 2.

Description: Number of oncogenes and tumor suppressors in individual gene groups defined by rank of BGRD width.

Title: Supplementary Data 3.

Description: Enrichment of oncogenes, tumor suppressors, housekeeping genes, or KEGG cancer pathways genes in individual gene groups defined by rank of H3K27me3 coverage in CD4+ T cell.

Title: Supplementary Data 4.

Description: Enrichment of oncogenes, tumor suppressors, housekeeping genes, or KEGG cancer pathways genes in individual gene groups defined by rank BGRD conservation level across the ENCODE (top panel) or Roadmap Epigenomic Project (bottom panel) samples.

Title: Supplementary Data 5.

Description: Enrichment of oncogenes in individual gene groups defined by rank of BGRD width in individual data sets of MCF10A (a), PrEC (b), HMEC(c) and HEM(d) samples.

Title: Supplementary Data 6.

Description: Enrichment of oncogenes and KEGG cancer pathways genes in individual gene groups rank by change of BGRD length and change of expression in cancer cells relative to matched non-cancer cells.

Title: Supplementary Data 7.

Description: The complete list of datasets analyzed in this manuscript.

Title: Supplementary Data 8.

Description: Gene name of TUSON oncogenes, TUSON tumor suppressors, housekeeping genes, and KEGG cancer genes.

Title: Supplementary Data 9.

Description: The width of H3K27me3 at individual genes in individual samples.

Title: Supplementary Data 10.

Description: gRNA sequences of candidate tumor-promoting genes and lncRNAs.

Title: Supplementary Data 11.

Description: PCR primers in Mutagenesis assay. Supplementary Data 12. Sequences of PCR primers in mutagenesis assay and realtime PCR primers for candidate tumor-promoting lncRNAs.
