# Supplementary material for: Broad genic repression domains signify enhanced silencing of oncogenes
Source: Nat Commun. 2020 Nov 3;11:5560. doi: 10.1038/s41467-020-18913-8 (PMC7641226; doi:10.1038/s41467-020-18913-8)
Supplement: Supplementary file 15 — Reporting Summary [file 41467_2020_18913_MOESM15_ESM.pdf]

nature research

Corresponding author(s): Kaifu Chen, Qi Cao, Lili Zhang

Last updated by author(s): 09/10/2020

Reporting Summary

Nature Research wishes to improve the reproducibility of the work that we publish. This form provides structure for consistency and transparency in reporting. For further information on Nature Research policies, see Authors & Referees and the Editorial Policy Checklist .

Statistics

For all statistical analyses, confirm that the following items are present in the figure legend, table legend, main text, or Methods section.

n/a	Confirmed	
☐	☒	The exact sample size (*n*) for each experimental group/condition, given as a discrete number and unit of measurement
☐	☒	A statement on whether measurements were taken from distinct samples or whether the same sample was measured repeatedly
☐	☒	The statistical test(s) used AND whether they are one- or two-sided<br>*Only common tests should be described solely by name; describe more complex techniques in the Methods section.*
☒	☐	A description of all covariates tested
☐	☒	A description of any assumptions or corrections, such as tests of normality and adjustment for multiple comparisons
☐	☒	A full description of the statistical parameters including central tendency (e.g. means) or other basic estimates (e.g. regression coefficient) AND variation (e.g. standard deviation) or associated estimates of uncertainty (e.g. confidence intervals)
☐	☒	For null hypothesis testing, the test statistic (e.g. *F*, *t*, *r*) with confidence intervals, effect sizes, degrees of freedom and *P* value noted<br>*Give P values as exact values whenever suitable.*
☒	☐	For Bayesian analysis, information on the choice of priors and Markov chain Monte Carlo settings
☒	☐	For hierarchical and complex designs, identification of the appropriate level for tests and full reporting of outcomes
☐	☒	Estimates of effect sizes (e.g. Cohen's *d*, Pearson's *r*), indicating how they were calculated

Our web collection on statistics for biologists contains articles on many of the points above.

Software and code

Policy information about availability of computer code

Data collection	All RNA-seq, microarray, and ChIP-Seq data were collected from ENCODE, GEO and NHLBI databases with accession ID. Human reference genome sequence version hg19 were downloaded from the UCSC Genome Browser website (https://genome.ucsc.edu). The reference gene list is downloaded from the website of the bioinformatics tool GREAT (http://great.stanford.edu/public/html/). 1500 oncogenes and 1500 tumor suppressor genes were collected from a recent publication (Davoli, T. et al. Cell. 2013). 330 genes in the Pathways In Cancer (hsa05200) were collected from the Kyoto Encyclopedia of Genes and Genomes (KEGG) database (https://www.genome.jp/kegg/).
Data analysis	All data were analyzed by perl(version 5.22.1), python(version 2.7.11) or R(version 3.2.1) scripts. All scripts were deposited into "figshare" website https://figshare.com/projects/SuperRepressiveDomain/59723. KEGG pathway analyses were performed by DAVID database version 6.8. RNA-Seq raw reads were mapped to the human genome version hg19 using TopHat version 2.1.1. Expression value (FPKM) for each gene was determined by the function Cuffdiff in Cufflinks version 2.2.1. Hg18 was converted to the hg19 version of the human genome using the tool LiftOver on UCSC https://genome.ucsc.edu/cgi-bin/hgLiftOver. DANPOS version 2.2.2 was employed to analyze ChIP-Seq data. The ChIP-Seq signal values at individual base pairs across individual genes were submitted to the software MeV version 4.8.1 to draw heat maps. The Dregion in DANPOS stored the signal value at each base pair in a Wiggle format file, which we next converted to bigWig format using the tool WigToBigWig (https://www.encodeproject.org/software/wigtobigwig/).

For manuscripts utilizing custom algorithms or software that are central to the research but not yet described in published literature, software must be made available to editors/reviewers. We strongly encourage code deposition in a community repository (e.g. GitHub). See the Nature Research guidelines for submitting code & software for further information.

Data

Policy information about availability of data

All manuscripts must include a data availability statement. This statement should provide the following information, where applicable:
- Accession codes, unique identifiers, or web links for publicly available datasets
- A list of figures that have associated raw data
- A description of any restrictions on data availability

The source data analyzed in this project are provided with this paper that are listed in the Supplementary Data 7. Important processed data were deposited to https://figshare.com/projects/SuperRepressiveDomain/59723.

Field-specific reporting

Please select the one below that is the best fit for your research. If you are not sure, read the appropriate sections before making your selection.

☒ Life sciences ☐ Behavioural & social sciences ☐ Ecological, evolutionary & environmental sciences

For a reference copy of the document with all sections, see nature.com/documents/nr-reporting-summary-flat.pdf

Life sciences study design

All studies must disclose on these points even when the disclosure is negative.

Sample size	All the experiment were performed in cell line. 3-6 individual samples were performed in each group. The sample size was mainly determined by our preliminary experiment. First of all, The sample size we chose need to provide sufficient statistical power. Cost, time and convenience of collecting the data were also taken into consideration.
Data exclusions	No data were excluded.
Replication	All experiments have been repeated for at least 3 times. All experiments were reproduced to reliably support conclusions stated in the manuscript.
Randomization	Cells in different groups were passaged from the same cell line and randomly assigned to experimental groups. All experiment results from individual samples are reported.
Blinding	After cell treatment, different groups were labeled with numbers only. During the data collection and analysis, the investigators were blinded to group allocation.

Reporting for specific materials, systems and methods

We require information from authors about some types of materials, experimental systems and methods used in many studies. Here, indicate whether each material, system or method listed is relevant to your study. If you are not sure if a list item applies to your research, read the appropriate section before selecting a response.

Materials & experimental systems

n/a	Involved in the study	
☐	☒	Antibodies
☐	☒	Eukaryotic cell lines
☒	☐	Palaeontology
☒	☐	Animals and other organisms
☒	☐	Human research participants
☒	☐	Clinical data

Methods

n/a	Involved in the study	
☒	☐	ChIP-seq
☒	☐	Flow cytometry
☒	☐	MRI-based neuroimaging

Antibodies

Antibodies used

All antibodies are commercially available and have been tested for the species used in this manuscript. The following antibodies were used:
1. CDC42BPA, 1:1000 dilution, Catalog # TA808223S, Origene
2. ANK2, 1:500 dilution, Catalog # 33-3700, Invitrogen
3. EXOC4, 1:1000 dilution, Catalog # TA811243S, Origene
4. BBX, 1:500 dilution, Catalog # NBP1-71905, NOVUS
5. RALGPS1, 1:500 dilution, Catalog # 13368-1-AP, Proteintech
6. VSNL1, 1:500 dilution, Catalog # UM870035, Origene

	7. GAPDH, 1:4000 dilution, Catalog # sc-32233, Santa cruz
Validation	1. CDC42BPA and EXOC4 antibody were validated by Origene. They transfected over expression vector (pCMV6-ENTRY EXOC4 and CDC42BPA) and control vector (pCMV6-ENTRY control) into HEK293T cell for 48h, then lysed the cell for western blot. EXOC4 and CDC42BPA over expression group shows the right western bands, while control group doesn't.<br>2. VSNL1 antibody was validated by Origene by western. They run western blot for human tissue lysates (Uterus, Breast, Brain, Liver, Ovary and Colon), and both shows the right bands.<br>3. ANK2 antibody was validated by Invitrogene by western. They run western blot for human SH-SK5Y cell line, which shows the right bands. There are also three other publications verified this antibody using ICC and IHC.<br>4. BBX antibody was verified by NOVUS. The immunogen for this product maps to a region between residue 841 and 891 of human Bobby Sox Homolog using the numbering given in entry NP_001136040.1 (GeneID 56987). The company verified the antibody by western and IP in Hela cells.<br>5. RALGPS1 antibody was verified by Proteintech. 13368-1-AP targets RALGPS1 in WB, ELISA applications and shows reactivity with human, mouse samples.<br>6. GAPDH antibody was verified by Santa cruz. Western bolt was performed in 293T Lysate, Jurkat whole cell lysate and Hep G2 cell lysate.

Eukaryotic cell lines

Policy information about cell lines

Cell line source(s)	Human breast cancer cell line MDA-MB-231, human prostate cancer cell line LNCaP and HEK293T cell line were purchased from ATCC
Authentication	MDA-MB-231, LNCaP and HEK293T cell line authentication were performed by ATCC. The authentication methods including: 1)Morphology check by microscope, 2)Growth curve analysis, 3)Species verification by Isoenzymology, 4)Identity verification with DNA fingerprinting for human cell lines
Mycoplasma contamination	MDA-MB-231, LNCaP and HEK293T cell lines were mycoplasma negative during routine tests
Commonly misidentified lines (See ICLAC register)	No commonly misidentified cell lines were used in the study.
